# Multipolar Atom Types from Theory and Statistical Clustering (MATTS) Data Bank: Impact of Surrounding Atoms on Electron Density from Cluster Analysis

**DOI:** 10.1021/acs.jcim.2c00145

**Published:** 2022-08-09

**Authors:** Paulina Maria Rybicka, Marta Kulik, Michał Leszek Chodkiewicz, Paulina Maria Dominiak

**Affiliations:** Biological and Chemical Research Centre, Department of Chemistry, University of Warsaw, ul. Żwirki i Wigury 101, 02-089 Warszawa, Poland

## Abstract

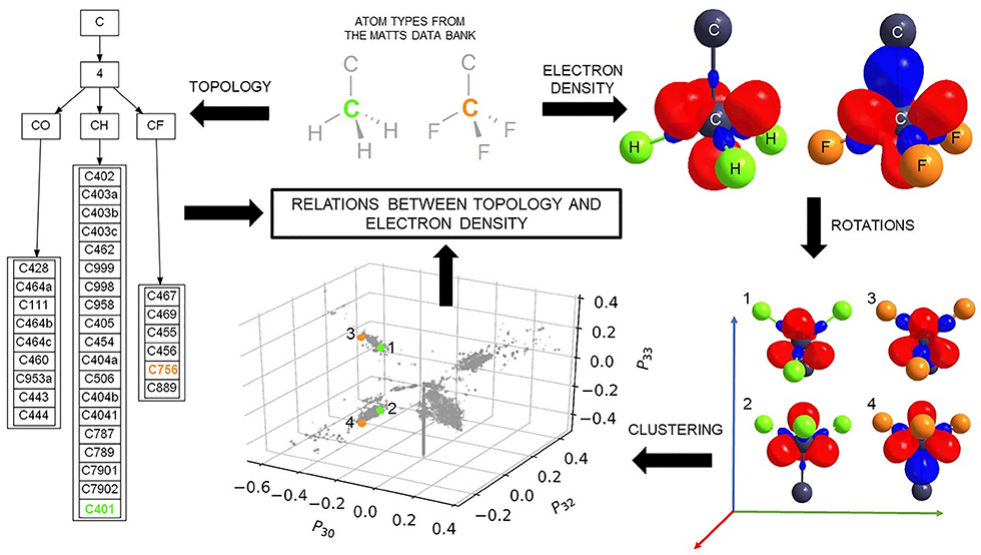

The multipole model (MM) uses an aspherical approach to describe electron density and can be used to interpret data from X-ray diffraction in a more accurate manner than using the spherical approximation. The MATTS (multipolar atom types from theory and statistical clustering) data bank gathers MM parameters specific for atom types in proteins, nucleic acids, and organic molecules. However, it was not fully understood how the electron density of particular atoms responds to their surroundings and which factors describe the electron density in molecules within the MM. In this work, by applying clustering using descriptors available in the MATTS data bank, that is, topology and multipole parameters, we found the topology features with the biggest impact on the multipole parameters: the element of the central atom, the number of first neighbors, and planarity of the group. The similarities in the spatial distribution of electron density between and within atom type classes revealed distinct and unique atom types. The quality of existing types can be improved by adding better parametrization, definitions, and local coordinate systems. Future development of the MATTS data bank should lead to a wider range of atom types necessary to construct the electron density of any molecule.

## 1 Introduction

The interpretation of the charge density distribution from single-crystal X-ray diffraction is now one of the most essential and useful tools in modern crystallography and has been widely used since its development.^1^ Access to an accurate electron density distribution enables the determination of different molecular properties, such as three-dimensional structure or one-electron properties, and provides information on molecular interactions. However, determining the high-resolution charge density distribution from an experiment is a time-consuming and complex procedure. Every so often it can even be unachievable, for example, because of the inability to obtain good-quality data from a crystal, experimental errors, limitations of electron density modeling, a lack of accurate phases, and major uncertainties in the hydrogen-atom positions and thermal motion.

Instead of measuring experimentally, electron density can be calculated using theoretical methods of quantum mechanics, based either on a wave function (Hartree–Fock and post-Hartree–Fock methods) or electron density (density functional theory—DFT). With methods based on wave functions, electron density is just one of the system properties derived from the wave function. On the contrary, in DFT, the electron density is the fundamental concept. Through the use of functionals of the electron density, other properties of the system can be computed, without the need for a wave function. For small molecules, calculating electron density with quantum mechanics is relatively fast and accessible but becomes challenging for macromolecules, crystals, or large-scale high-throughput analyses.

Intending to get past limitations with the experimental approach, the idea of transferability of parameters describing electron density between chemically related molecules was introduced to crystallography by Brock et al. in 1991 and has since been used to create data banks of aspherical atom parameters.^2^ There are three well-established databases: the Invariom database,^3,4^ the experimental library of multipolar atom model (ELMAM),^5,6^ and the University at Buffalo Data Bank (UBDB).^7−10^ Lately, the UBDB was superseded by the multipolar atom types from theory and statistical clustering (MATTS) data bank.^11^ Pseudoatom data banks allow the replacement of independent atom model (IAM) scattering factors, typically used for refining crystal structures, with aspherical ones calculated from the transferable aspherical atom model (TAAM). In effect, the TAAM refinement increases the accuracy and precision of defining thermal atomic displacement parameters and molecular geometry, particularly for hydrogen atoms.^12,13,19^ Pseudoatom data banks consist of multipole model (MM) parameters that can be applied to reconstruct the electron density distribution of known molecules and to quickly derive various properties of electron density, such as electrostatic interaction energy, molecular electrostatic potentials, and dipoles.^14−24^ Overall, pseudoatom data banks can be applied to study crystals of small-molecule compounds, nucleic acids, and proteins (e.g., neuraminidase with around 350–400 amino acids, syntenin PDZ2 domains with 90 amino acids,^9^ and protein kinase complexes with around 300–350 amino acids^19^) and to predict properties of unknown ones as molecular electron density determines all their properties. Apart from X-ray diffraction, the pseudoatom data banks were implemented for electron diffraction (3D ED and microED) to refine structures with multipolar electron scattering factors.^20−47^ Electron scattering factors of charged atoms can be also used for refinement of atomic models against single-particle cryo-electron microscopy (cryo-EM) maps.^22^

The purpose of the MATTS data bank is to recreate the electron density with sufficient quality for crystallography, structural biology, or chemistry. The quality of the properties calculated from this electron density should be much better than that given by force fields but it does not have to be as high as from quantum mechanics. Thus, the MATTS data bank approach is placed between molecular mechanics and quantum mechanics—it is more accurate than molecular mechanics and faster than routinely used quantum mechanics calculations.

**Figure 1:**
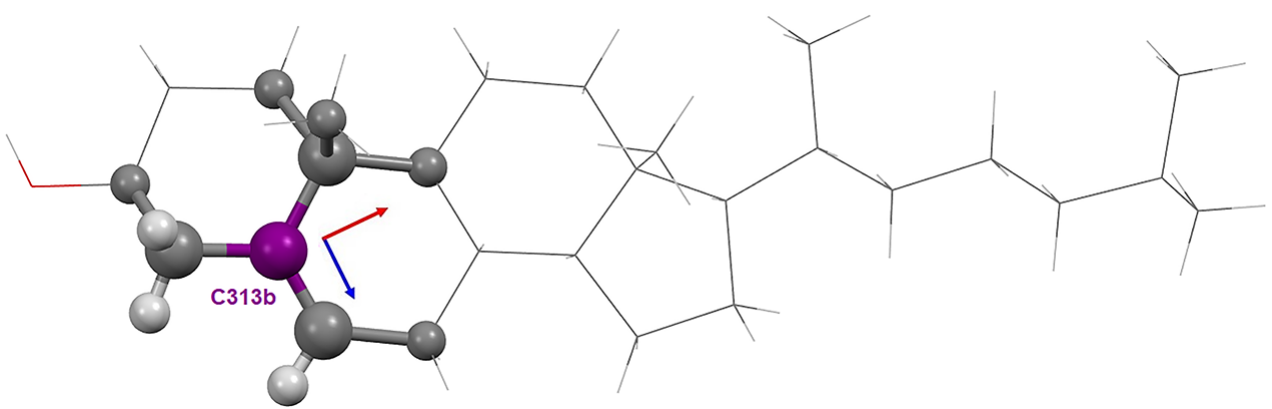
Atom type C313b in the structure of cholesterol.^26^ The definition in the MATTS data bank for this atom type includes the central atom (in purple) and the first and the second covalently bonded neighbors, marked with thick bonds and atom colors. The bond along the *Z*-axis (blue) is double. The exact definition of atom type C313b can be found in the Supporting Information (Table S1).

**Figure 2:**
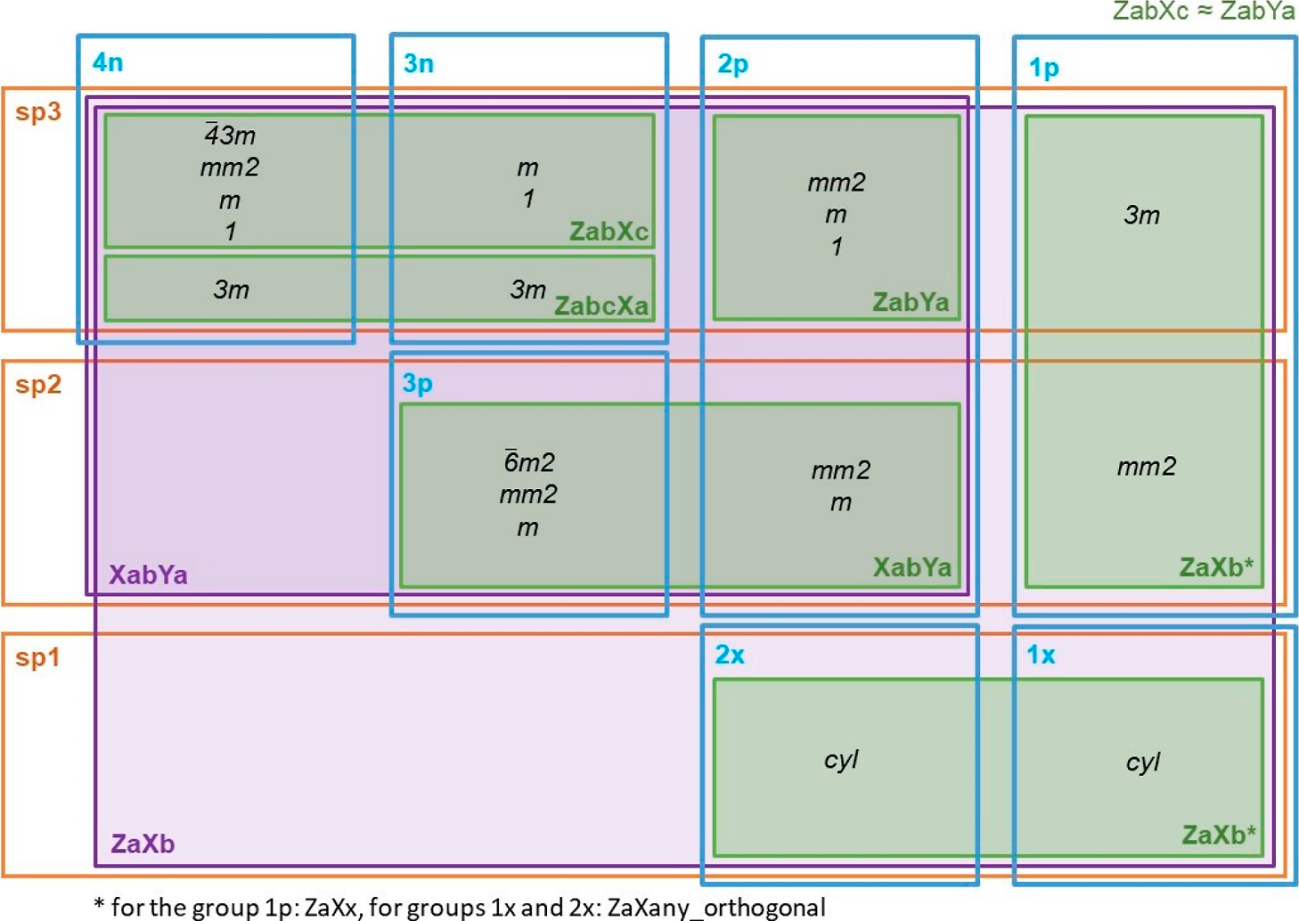
Possible symmetries and local coordinate systems based on hybridization (sp^3^, sp^2^, and sp^1^) and group (1x, 1p, 2x, 2p, 3p, 3n, and 4n). Green indicates the minimum set of local coordinate systems required to see all possible symmetries. Purple indicates the local coordinate systems which are necessary to cluster and compare atom types from different groups.

For the bank to work properly, a well-designed algorithm for defining the types of atoms is necessary. In the MATTS data bank, the parameters describing aspherical pseudoatom types are obtained by Fourier space fitting to molecular electron density distributions given by quantum chemical calculations in the vacuum. A different approach to the assignment of atom types is used in force fields where main focus is on energy calculation and geometry optimization. The general AMBER force field (GAFF) is widely used since its development by Wang et al. in 2004 and characterizes atom types commonly occurring in organic molecules.^23^ Both in the MATTS data bank and GAFF, atom types are described using a set of various descriptors of chemical character that can be assigned to specific hybridizations and aromatic or aliphatic chemical groups; however, some differences are also present. In the MATTS data bank, for example, there is no need to define whether the bond is single, double, or triple because it can be inferred from the local connectivity of the atoms in the vicinity (see Figure 1). A molecule which is difficult to parametrize with GAFF/GAFF2 atom types is shown in Figure 2f by Mobley et al.^25^ In our system of atom type assignment, this problem and similar problems do not exist, and only two atom types—C330 and C332 are necessary to fully describe all carbon atoms in this molecule. In the force fields, the bond order is often the part of the atom type definition. The force field approach is designed to provide an estimate of the overall interaction energy, but it is not tuned to adequately represent individual energy components.^24^ Also, there are some systematic errors in the GAFF parameters, for example, for alcohols and alkenes.^25^

The MATTS data bank is based on the Hansen–Coppens formalism that presents an aspherical approach to electron density modeling.^27^ The electron density of a molecule or a crystal is divided into contributions from individual atoms, and each atom is represented by a pseudoatom. The pseudoatom density contains three components, a spherical core, spherical valence, and deformation valence, which is described using the sum of atom-centered real spherical harmonics (*d*_*lm*_)
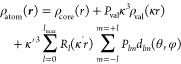
1where ρ_core_ and ρ_val_ are spherical core and spherical valence electron densities, the multipliers *P*_val_ and *P*_*lm*_ are the populations of valence electrons and deformation functions, and the coefficients κ and κ′ are scaling parameters that describe the contraction–expansion of the spherical and deformation valence electrons. Real spherical harmonics *d*_*lm*_ are defined in the local coordinate system, and the values of *P*_*lm*_ parameters depend on the orientation of the said system. It is convenient to orient the local coordinate system with respect to the positions of the closest atoms.

The MATTS data bank, being a descendant of the UBDB, redefines numerous atom types and introduces new ones. Up to date, there have been no attempts to draw general conclusions on the electron density–atom type relations in the data bank. Currently, due to the large number of atom types introduced into the data bank, such an analysis appears to be possible and necessary. The main idea behind this work was to compare the atom types currently deposited in the data bank and discover similarities in the spatial distribution of electron density for them. Such an approach makes it possible to distinguish between general and specific atom types, matching the level of information to the needs of a user and define new, more universal atom types that describe a wider range of atoms. Additionally, the information in the data bank can be viewed not only from the point of the electron density described using multipole parameters but also from the point of topology defined by a neighborhood of the central atom. The similarities within and between these two approaches can be easily found by using clustering methods that allow us to create groups of similar atom types by using different criteria. It is essential to keep in mind that the electron density in the aspherical approach given using eq 1 is described by a collection of parameters (κ, *P*_val_, κ′, and *P*_*lm*_) that are not independent from one another. The main purpose of multidimensional clustering with every possible value of κ, *P*_val_, κ′, and *P*_*lm*_ is to get the whole image of electron density of an atom type and, additionally, to check for errors. Noteworthy, increasing the number of dimensions usually leads to various issues generally known as “the curse of dimensionality”.^28,29^ Multiple dimensions are challenging to think in, almost impossible to visualize, and the idea of distance becomes less precise as the number of dimensions grows, seeing as the distance between any two points in a given data set converges and data become sparse.

## 2 Methods

### 2.1 Data

All analyses were carried out using the MATTS2021 data bank that contains entries for 651 atom types^11^ of chemical elements including C, H, N, O, S, P, F, Cl, and Br. The atom types in the MATTS2021 data bank give information about their topology (chemical elements, numbers and types of neighbors, ring membership, the orientation of the coordinate system for the MM, etc.) and electron density (κ, *P*_val_, κ′, and *P*_*lm*_).

Each atom type is specified by its local chemical topology (molecular fragment) defined through the atomic connectivity list, where the central atom and its first, second, and further neighbors are defined. The atoms that are covalently bonded to the central atom are called the first neighbors. The second neighbors are not directly connected to the central atoms, but they are covalently attached to the first neighbors, whereas the third neighbors are attached to the second neighbors and so on. The level of neighbors up to which (first, second, etc.) the type is defined varies from one atom type to another. Each atom on the connectivity list might further be specified using the following descriptors: chemical element types, planarity of the group of atoms constituted from a given atom and its first neighbors, belonging to five-, six-, or/and seven-membered rings which are planar and are formed by planar groups, and belonging to three- or/and four-membered rings. To each atom type, a set of electron density parameters is assigned (κ, *P*_val_, κ′, and *P*_*lm*_) along with the definition of a local coordinate system in which multipolar functions were expressed. In addition, local symmetry, which has to be fulfilled by a given set of multipolar functions, is provided.

The values for density parameters of a given atom type result from averaging over all atoms found in a set of model molecules and belonging to that atom type. Only *P*_*lm*_ parameters larger than 0.002 e that fulfill the defined symmetry, whose values are also larger than one sample standard deviation, are stored in the data bank. Coordinates of model molecules are taken from the Cambridge Structural Database,^30^ and electron density is obtained from procedure described in the first publication about the MATTS data bank.^11^ For more details, especially regarding the procedure of defining atom types and bank making, see previous publications about the UBDB^10,31^ and MATTS data bank.^11^ The current version of the MATTS data bank can be downloaded from http://4xeden.uw.edu.pl/.

### 2.2 Rotation of Local Coordinate Systems and the Effect of Symmetry on *P*_*lm*_ Values

The importance of specifying a proper local coordinate system to orient multipole functions has been noted before in the literature.^32−35^ Atom types deposited in the MATTS data bank are defined in only one of many available arrangements of the local coordinate system (see Figure 1). Also, the assigned local coordinate systems differ between atom types. The values of *P*_*lm*_ parameters change upon rotations of local coordinate systems. For each atom type, *P*_*lm*_ parameters were recomputed in several orientations of local coordinate systems. Only selected orientations (rotations) were considered, and they were chosen by taking into account the number of first neighbors, possible orbital hybridizations of a given central atom, and possible local symmetries. It was not feasible to consider all orientations due to limited computational resources.

To orient the local coordinate system, two non-collinear directions have to be specified to orient two axes of the coordinate system, preferably in explicit relation to the bonds formed between the central atom and its first neighbors. The orientation of the third axis is based on the right hand rule and is perpendicular to both the first axis and the second axis. The MATTS data bank was divided into eight independent groups of atom types, 1x, 1p, 2x, 2p, 3p, 3n, 4n, and 6n (Table 1), depending on the number of the first neighbors and its relative orientation in space. For atom types with one first neighbor, the 1x group was created by choosing the atom types for which the first and second neighbors were collinear with the central atom, and it is impossible to unambiguously orient the local coordinate system with respect to their positions. The rest of the atom types having only one first neighbor were assigned to the 1p group. For atom types with two first neighbors, the dividing factor is the presence or absence of collinearity between the central atom and its first neighbors. If the three atoms are collinear, the atom type belongs to the 2x group. Otherwise, it belongs to the 2p group. Atom types with three first neighbors were divided into two groups, depending on if the central atom and its first neighbors lay on the same plane (3p) or not (3n). All atom types in the MATTS2021 data bank having four (4n) or six (6n) first neighbors are not planar and were not further divided. For the 6n group, only one rotation was considered as it contains only one atom type: phosphorus from the PF_6_^–^ molecule. Consideration of other possible rotations for 6n was unnecessary for the current version of the MATTS data bank.

**Table 1:** Number of Considered Rotations of Local Coordinate Systems for Every Group^a^

group	characteristic	no. of coordinate systems	no. of atom types	total
1x	1 neighbor, symmetry: cylindrical	1	29	29
1p	1 neighbor, symmetry: m	1	60	60
2x	2 neighbors, symmetry: cylindrical	2	5	10
2p	2 neighbors, symmetry: m, mm2, 1	6	68	408
3p	3 neighbors, planar	12	297	3564
3n	3 neighbors, not planar	16	27	432
4n	4 neighbors, not planar	40	164	6560
6n	6 neighbors, not planar	1	1	1
sum			651	11064

a No. of coordinate systems − total number of considered local coordinate systems for each atom type from the group. No. of atom types − number of atom types in the group. Total − total number of local coordinate systems calculated for the group.

The presence of lone electron pairs has to be taken into consideration when setting coordinate systems. Electron density of lone pairs may contribute to the population of some multipolar functions at a similar level as covalent bonds. To take into account all possible orientations of lone pairs, not only the first neighbor atoms but also possible hybridization of the central atoms must be considered. The initial assignment of possible hybridization to atom types was based on the number of first neighbors and planarity of the group (see Figure 2).

With a proper local coordinate system, the symmetry of electron density associated with a given individual atom and then with a given atom type will emerge from the values of *P*_*lm*_ parameters, without any previous arbitrary decisions being made on the basis of atom-type topology. For some symmetries to be easily detected, the local coordinate system has to be aligned with the symmetry elements. The complete set of rotations of the local coordinate system was designed to reveal every possible local symmetry. Possible local symmetries were predicted by considering the number of first neighbors and lone pairs, their relative orientation in space around the central atom (collinearity and planarity), and the assumption that some first neighbors might be regarded the same from an electron density point of view (even though they are different from the topology point of view).

With the local coordinate system being optimally oriented with respect to symmetry elements for a given point symmetry group, the site symmetrization of spherical harmonics (multipolar functions) is easily established since any point symmetry operation transforms a spherical harmonic into another one of the same order of l.^36^ A complete set of site-symmetric spherical harmonics can be obtained by applying rules corresponding to the group generators. Spherical harmonics not allowed by these rules should vanish, that is, *P*_*lm*_ populations associated with them should be equal to zero (see Table S2 in the Supporting Information). Proper orientation of the local coordinate system with respect to local symmetry is important not only to assign atom types to individual atoms in molecules but also for the atom type creation process. When *P*_*lm*_ parameters for individual atoms are averaged to create an atom type, the symmetry of atom electron density will be preserved only if the proper local coordinate systems are assigned. Otherwise some (or all) *P*_*lm*_ parameters will average out to zero values, falsely suggesting higher symmetry. Proper recognition of local symmetry of electron density for a given atom type would allow to decide whether, from an electron density point of view, some first neighbors are the same or not. If they were the same, there would have been no need to differentiate them from the topology point of view and atom type definition might have been generalized. Among many possible rotations, the one which allowed to observe a larger number of possible point symmetry groups was chosen. Next, the rotation that, for a defined hybridization, had one dominant (the highest occupied) multipole in all possible symmetries for a given group of neighbors was preferred over the combination of multipoles containing a few similarly occupied multipoles.

While orienting the local coordinate systems, as many first neighbors as possible should be engaged, so that the averaged electron density would not be biased by a better description of only a few neighbors. This is because the coordinate system is orthogonal but valence angles between the central atom and its first neighbors are not and may slightly change from one individual atom to the other. First neighbors considered to be symmetry-equivalent should be engaged in setting the coordinate system to the same extent.

It may happen that from the electron density point of view, some atom types might be similar to one another across the above defined six groups. To allow comparison (clustering of electron density parameters) across the groups, atom types belonging to various groups must still have the same rotation (orientation) of their coordinate system. Such rotations were added to the analysis.

Three combinations of the first and second axes of the local coordinate systems were taken into consideration: ZX, ZY, and XY. First neighbors are denoted as a, b, c, and d. The first axis was placed in a few different ways: from the central atom toward the neighboring first atom (which gives systems like ZaXb) or in average direction between two (ZabXc, ZabYa, and XabYa) or three (ZabcXa) neighbors. It should be noted that the ZaXb system applies to atom types with at least two first neighbors and all three atoms are not collinear. If an atom type does not meet this criterion, one of the alike systems is used: ZaXany_orthogonal, where three atoms are collinear (in groups 1x and 2x, and the system enforces cylindrical symmetry), or ZaXx if they are not (the 1p group, x means the second or further neighbor). For simplicity, both of these systems are referred to as ZaXb in the following text.

**Figure 3:**
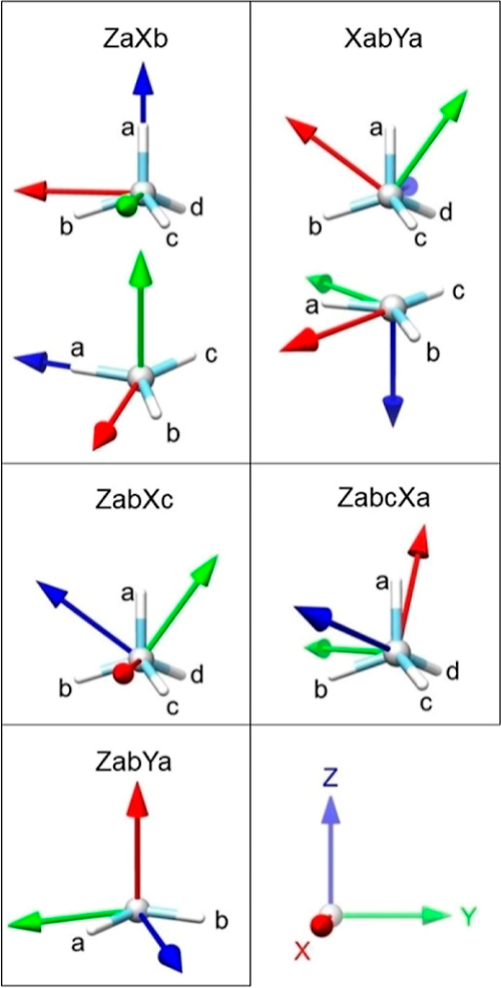
Examples for each of the considered local coordinate systems in planar and non-planar groups. All possibilities with their corresponding names can be found in the Supporting Information in Figure S1.

Examples of each of the possibilities are shown in Figure 3. Visualizations of all considered local coordinate systems can be found in the Supporting Information in Figure S1. The names of individual rotations are defined as follows: [group]_[rotation number]-[local coordinate system]. For example, 4n_62-ZabdXa equals “rotation number 62 for an atom type in the group 4n within the ZabcXa system, where the Z axis is in the average direction between atoms a, b, and d and the X axis is in the direction of atom a”.

**Figure 4:**
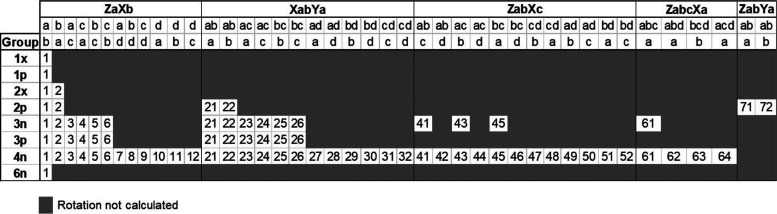
All considered rotations of local coordinate systems for every group with assigned numbers. First row: general name of the system, second row: the direction of the first axis, and third row: the direction of the second axis.

The ZaXb system (with ZaXx and ZaXany_orthogonal) includes rotations 1–12 (see Figure 4). Rotations are the result of finding all possible combinations of two neighbors out of the maximum four for atom types from the rotated groups. Figure 4 also shows the 6n group with six neighbors in PF_6_^–^, so the actual maximum number of neighbors is six; however, this group was not included in the process of rotating the local coordinate system. ZaXb is the most general system that describes every group and is the basis for comparing them with one another. It is also the only used system to describe 1x and 1p groups. In the ZaXb system, a few possible symmetries may appear for atom types in the 1p group, depending on the conditions met. The 3m symmetry occurs for atom types with sp^3^ hybridization and all three electron pairs treated as equal, whereas the mm2 symmetry is present for atom types with sp^2^ hybridization and two equivalent electron pairs. The XabYa system includes rotations 21–32 and is designed for atom types with sp^2^ hybridization, that is, for 2p and 3p groups. There are three possible point symmetry groups here: 6̅*m*2, *mm*2, or *m*. The 6̅*m*2 symmetry should appear only for the 3p group when all neighbors are the same (*a* = *b* = *c*). The *mm*2 symmetry should appear when two neighbors are the same (*a* = *b* or *a* = *c*), which is possible in both groups (2p and 3p). The m symmetry should always be present since these two groups are planar. The ZabXc system includes rotations 41–52 and was designed for 4n and 3n groups (hybridization sp^3^) with many possible symmetries: 4̅3*m*, *mm*2, *m*, or 1 (which indicates no symmetry). The 4̅3*m* symmetry should appear when *a* = *b* = *c* = *d*, that is, when all four neighbors are equal, the *mm*2 symmetry should appear when neighbors are two pairs of equal atoms (*a* = *b* and *c* = *d* or *a* = *c* and *b* = *d* or *a* = *d* and *b* = *c*), and m symmetry should appear when only two neighbors are equal (*a* = *b* or *a* = *c* or *b* = *c*, etc). The 4̅3*m* and *mm*2 symmetries should appear only for the 4n group, not 3n. The ZabcXa system includes rotations 61–64. The sole purpose of the ZabcXa local coordinate system is to check for the presence of 3m symmetry, which, for the 3n group, should be already revealed for the 3n_61-ZabcXa rotation where *a* = *b* = *c* (all three neighbors are identical). Likewise, for the 4n group, 4n_61-ZabcXa, 4n_62-ZabdXa, 4n_63-ZbcdXb, and 4n_64-ZacdXa are enough to cover all possibilities of threefold axis distinctive orientation for this group. The ZabYa system includes rotations 71 and 72. It was introduced especially for the 2p group with the sp^3^ hybridization, which does not have the third (c) neighbor, to nevertheless achieve analogous orientation of multipolar functions like in the case of ZabXc rotation for 4n and 3n groups, the last with the sp3 hybridization. For the 2p group, there are two possible symmetries: *mm*2 or m. The *mm*2 symmetry will be revealed when *a* = *b*, otherwise the m symmetry should be present (assuming sp^3^ hybridization and that the two electron pairs are equivalent).

During the rotation of local coordinate systems, we did not want to enforce any symmetry higher than 1; thus, the symmetry filter was set to “no”, and no filtering by symmetry was applied to *P*_*lm*_ parameters. Atom types for which at least one *P*_*lm*_ parameter appeared to have sample standard deviation larger than 0.05 e were assigned a flag “inconsistent”. The code and computational details of the procedure used for topology clustering can be found in the Supporting Information.

### 2.3 Topology Clustering

Topology clustering determined relations between atom types and their neighbors by creating a hierarchical data structure that is later in the text referred to as ‘a tree’. The clustering was based on topology descriptors such as the element type of the central atom, first and second neighbors, and the number of the first and second neighbors. The first level of a tree determines the chemical element of the considered group of atom types and is the first dividing criterion. Next levels take into account information about the number of first neighbors, types of first neighbors, and also, if needed, the number and types of second neighbors, which gives the second, the third, the fourth, and the fifth level of a tree, respectively. The terminal level of a tree includes names of atom types described by one branch of a tree. Such a system of classification was our arbitrary choice based on previous experience gained from the manual building of the bank. Topology descriptors such as group planarity or belonging to rings were not used as levels’ dividers.

**Figure 5:**
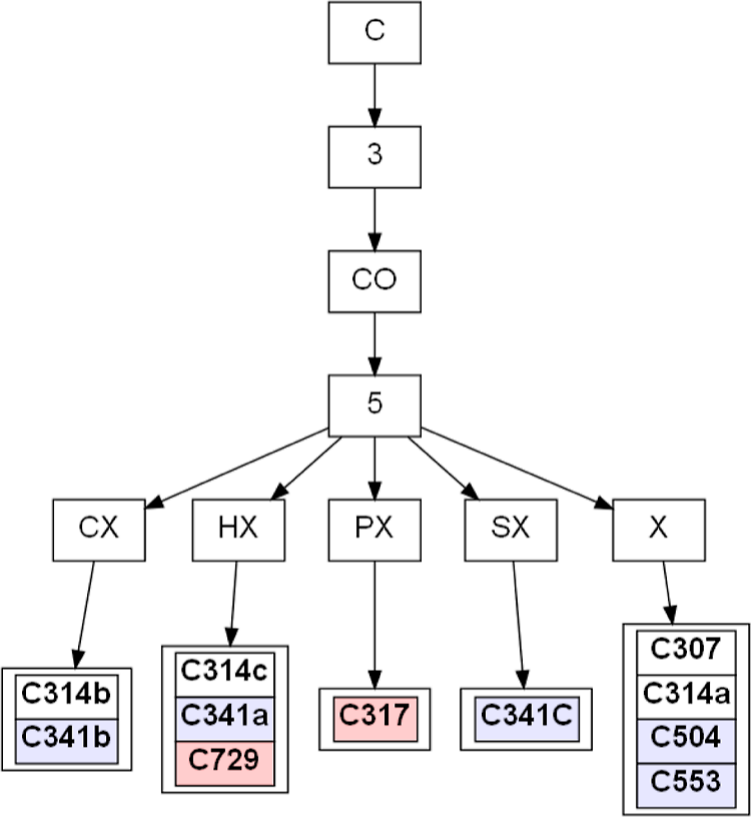
Exemplary branch from an extended tree. From top to bottom: the chemical element of a central atom, the number of first neighbors, chemical elements of first neighbors, the number of second neighbors, chemical elements of second neighbors (X—any element), and names of atom types in the MATTS2021 data bank. The bold font indicates that an atom type belongs to a planar group. The size of the ring is specified by background colors, and its description can be found in Figure S3.

Topology clustering used for visualizing atom types can be easily adapted to the needs of the user, for example, by excluding information about the second neighbors, dividing trees by the number of first neighbors or element types, or including additional information in the terminal node. There are three different structures of trees used in this work, adapted to the analyzed properties of atom types. An *extended tree* shows topology in detail with information about the number and chemical element types of the first and second neighbors and about planar properties and belongingness to a ring. An exemplary branch from an extended tree is shown in Figure 5. A *general tree* does not include details on second neighbors and is used to connect electron density and topology clustering using a colored background coding information about density clusters. Finally, a *concise tree* has a purpose similar to that of the general tree but compresses the information about the size of electron density clusters even more by excluding element types of the first neighbors and names of atom types. Schemes of all structures of trees are shown in Figure S2 in the Supporting Information. The code and computational details of the procedure used for topology clustering can be found in the Supporting Information.

### 2.4 Electron Density Clustering

**Figure 6:**
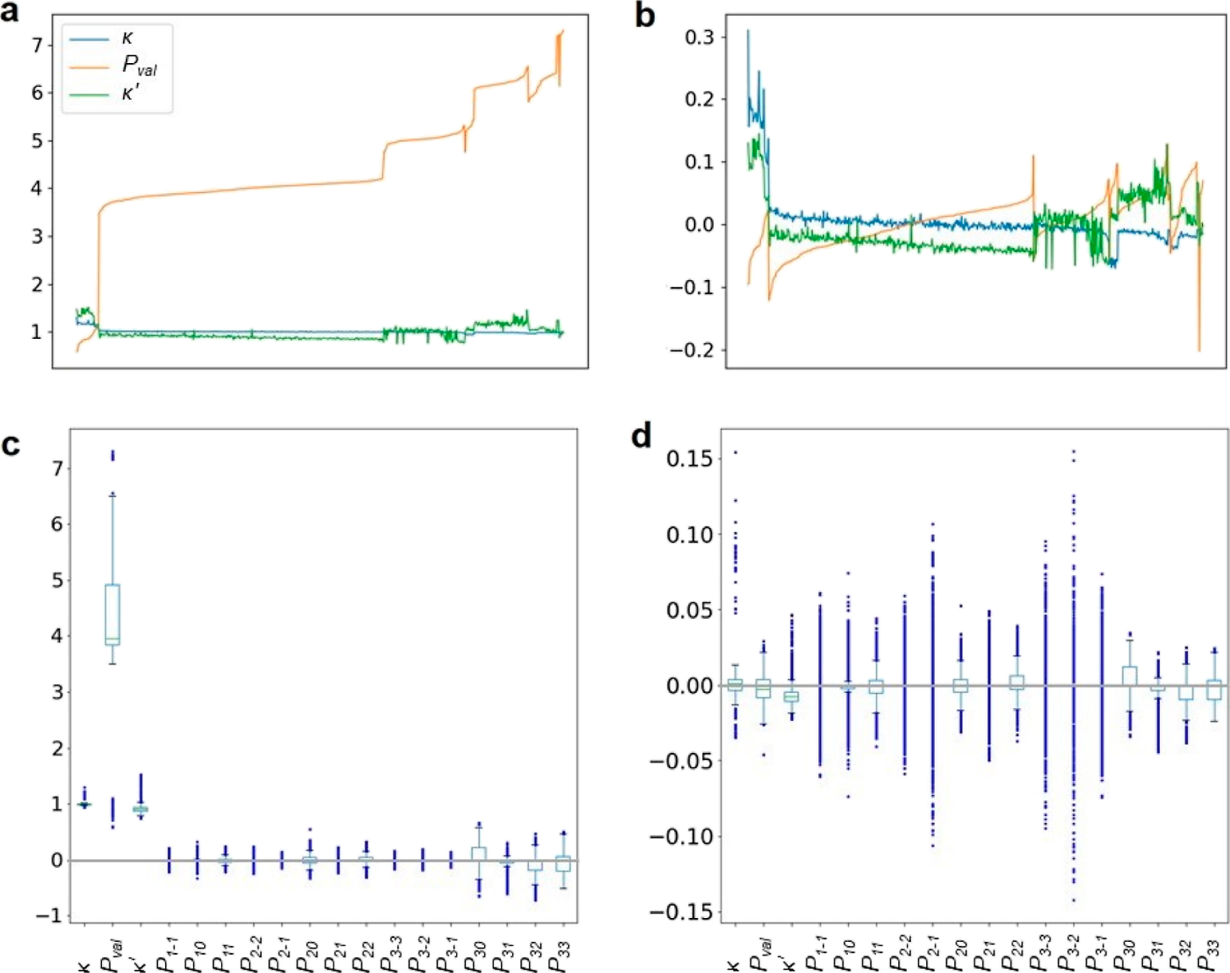
First row: κ, *P*_val_, and κ′ values for original (a) and subtracted and normalized data (b). Atom types were sorted increasingly based on the number of valence electrons (H, C, N, P, O, S, F, Cl, and Br) and then using *P*_val_ from the lowest to the highest for each element. Second row: box plots showing the distribution of all parameters for original (c) and subtracted and normalized data (d).

Although all *P*_*lm*_ parameters have fairly comparable values, both positive and negative around 0, κ, *P*_val_, κ′ are different: κ and κ′ have always positive values around 1, and *P*_val_ parameters have also positive values that oscillate around the formal number of valence electrons specific for an element of the atom type, ranging from 1 to 7 (see Figure 6a,c). Usually, it is advised to bring all analyzed parameters to the same scale before clustering. Possible modifications of the original parameters from the MATTS data bank include subtraction and normalization, and their purpose is to mostly limit the domination of *P*_val_. By subtracting 1 from κ and κ′, the new values around 0, both positive and negative, are obtained. Subtracting the number of valence electrons (four for carbon, five for nitrogen, six for oxygen, etc.) from *P*_val_ gives values around 0, both positive and negative. Normalization is achieved by dividing original values of parameters by the L2 norm, which is calculated as the square root of the sum of the squared values of one of κ, *P*_val_, κ′, or *P*_*lm*_ parameters for all 11064 generated entries for atom types.^37^ The normalized vector is given using eq 2
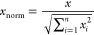
2where *x* is the vector of covariates (one of κ, *P*_val_, κ′, or *P*_*lm*_ parameters) of length n, and in our case, *n* = 11 064 (the number of total possibilities of atom types rotated in local coordinate systems).

Density clustering of atom types using electron density parameters was performed using DBSCAN (density-based spatial clustering and application with noise), which is a partitioning method that had been introduced by Ester et al. in 1996.^38^ It can distinguish clusters of various sizes and shapes for complicated data with noise and outliers. The key idea behind DBSCAN is that each point in the cluster needs to have at least the specified minimum number of adjacent points (*MinPts*) within a given radius epsilon (Eps). There were 18 dimensions taken into account: κ, *P*_val_, κ′, and 15 different *P*_*lm*_ for corresponding multipoles (*P*_1–1_, *P*_10_, *P*_11_, *P*_2–2_, *P*_2–1_, *P*_20_, *P*_21_, *P*_22_, *P*_3–3_, *P*_3–2_, *P*_3–1_, *P*_30_, *P*_31_, *P*_32_, and *P*_33_). The electron density in the MATTS data bank is described up to hexadecapoles, that is, *l* = 4. However, including values for hexadecapoles would increase the number of dimensions by 9, so they were omitted in the analysis of electron density to simplify the calculations and interpretation of results. A correct *Eps* parameter required to perform DBSCAN can be arbitrary provided by the user or estimated using ‘the knee/the elbow method’.^39,40^ The method calculates the distance between a point and its nearest neighbors, whose number can be specified manually, chooses the smallest one, repeats the steps for all points, makes a plot of minimum distances, and finds “a knee” or “an elbow” of the plot that suggests where the optimal *Eps* value is. The code and computational details of the procedure used for electron density clustering can be found in the Supporting Information.

#### 2.4.1 Multi-Leveled Utility Approach to Density Clustering

The original values of parameters such as κ, *P*_val_, κ′, and *P*_*lm*_ are needed to interpret the electron density in a beneficial way without losing information about their correlations with one another and differences in their distribution. For the original data set, the minimum number of points (*MinPts*) for the DBSCAN was adjusted to 2. Changing *MinPts* to 3 resulted in the absence of two-element clusters and a major increase in the number of outliers (about 40% of all atom types). On the other hand, *MinPts* = 1 resulted in an absence of outliers and tens of one-element clusters. The *Eps* parameter was found automatically by the knee locator method and set as 0.174. The main output that provides information is a table with clustered atoms, where each cluster is labeled with a different number and outliers are marked as “-1”. DBSCAN performed for *Eps* = 0.174, which is general for the entire MATTS2021 data bank with diverse atom types, gives a considerable number of 92 clusters and 21 outliers. The created clusters contain atom types whose parameters are fairly similar, within the given *Eps* range. After the initial clustering, 2 clusters out of 92 included atom types of more than one chemical element, combinations being: O and S (cluster 2) or Br and Cl (cluster 5). Also, 3 out of 92 clusters included atom types from different groups: previously mentioned cluster 2 (O 1p, 2p, 3n, and S 2p), cluster 9 (N 2p, 3n, and 3p), and cluster 10 (C 3n, 3p, and 4n). The implication that such a cluster should be further divided is revealed in calculated standard sample deviations for κ, *P*_val_, κ′,and *P*_*lm*_ parameters in a given cluster when even one of them is larger than 0.05. The *Eps* = 0.174 was not sufficient enough to divide these clusters. Thus, DBSCAN was performed again many times for individual clusters using decreasing values of the *Eps* parameter for each level (see Table 2). Usually, *Eps* automatically found for each cluster independently was good enough to fulfill its purpose of creating reasonable sub-clusters of atom types. However, in some cases, manually checked *Eps*, with minimal deviations from the original value chosen using the algorithm, would create sub-clusters with sample standard deviation below 0.05. For example, in the case of S atom types from the 3n group, automatically found *Eps* for clusters 12, 13, and 14 was 0.173 in all three cases, which was too similar to the one for the entire data set and would not divide these clusters. Thus, other *Eps* values were checked manually to see if any of the S atom types would separate itself from others as sample standard deviation was larger than 0.05 and relations between the atom types were not clear. Also, for cluster number 20, and later 20_0, *Eps* had to be chosen manually as ‘a knee’ algorithm was not working properly because all rotations in these clusters were for atom types with topologically indistinguishable first neighbors. Thus, each rotation had a corresponding rotation with the exact same values of *P*_*lm*_ parameters and all distances between data points, which are necessary for finding *Eps* by ‘a knee’ method, were zeros. The final label of a created cluster is a compilation of labels from all levels of clustering joined by the underscore symbol. An Excel file with full-density clustering results and their statistical analysis can be found in the Supporting Information as an individual file named ‘density-clustering.xlsx’.

**Table 2:** Eps Values Used in the Next Levels of DBSCAN, the Ones Marked with * were Found Manually

chemical element of atom types	group	coordinate system	divided cluster	*Eps*
C	3n, 3p, 4n	ZaXb, XabYa, ZabXc, ZabcXa	10	0.137
	3n, 3p, 4n	ZaXb, ZabXc, ZabcXa	10_0	0.111
	3n, 3p	ZaXb	10_0_0	0.095
	4n	ZaXb, ZabXc	10_0_16	0.086
	3n, 3p, 4n	XabYa	10_1	0.127
	3n, 3p, 4n	XabYa	10_1_0	0.120
	4n	ZabXc	20	0.110*
	4n	ZabXc	20_0	0.080*
N	2p, 3n, 3p	ZaXb, ZabYa, XabYa, ZabXc, ZabcXa	9	0.085
O	1p, 2p, 3n	ZaXx, ZabYa, ZaXb, XabYa, ZabXc, ZabcXa	2	0.109
	1p, 2p, 3n	ZaXx, ZabYa, ZaXb, XabYa	2_0	0.087
	1p, 2p	ZaXx, ZabYa	2_0_1	0.071
	2p, 3n	ZaXb	2_0_3	0.031
	2p, 3n	XabYa	2_3	0.071
S	2p	ZaXb, XabYa, ZabYa	2	0.109
	3n	ZaXb	12	0.155*
	3n	XabYa	13	0.155*
	3n	ZabXc	14	0.004*
	4n	ZaXb	80	0.065
	4n	XabYa	81	0.050
	4n	ZabXc	82	0.075
	4n	ZabcXa	83	0.052

As the clustering progressed, we realized that the clusters derived from the initially largest clusters, that is, 10_0_0_0 and 10_1_0_0, will still sporadically have at least one sample standard deviation above 0.05 and decided to divide the clusters up to four levels only. In summary, clusters created by initial clustering were divided into sub-clusters by the next levels of clustering with the primary purpose of decreasing sample standard deviation values for *P*_*lm*_ parameters below 0.05. Sample standard deviations for κ, *P*_val_, and κ′ were not considered as a criterion for further division of clusters as these parameters remained unchanged during the process of rotation. Sporadically, we did additional levels of clustering, even though sample standard deviation did not indicate the necessity to do so, to check if some previously unseen connections would appear. In some cases, especially carbon atom types, clustering beyond four levels would be pointless as sample standard deviation for at least one new sub-cluster would remain above 0.05.

### 2.5 Data Presentation/Visualization

Chimera 1.15rc^41^ was used for visualization of rotations in local coordinate systems. WinXD 1.05^42^ and MoleCoolQt 4.8.6^43^ were used for visualization of multipolar functions. Mercury 4.1.0 was used for visualization of the cholesterol molecule.^44^

## 3 Results

Each method chosen to cluster properties of atom types gives different results. Topology clustering takes into account information about neighbors and planarity, arranging atom types in a structured way. Density clustering interprets parameters essential to model electron density using the Hansen–Coppens formalism, that is, κ, *P*_val_, κ′, and *P*_*lm*_, considers all possible arrangements of local coordinate systems, and returns a set of clusters.

### 3.1 Topology Clustering

The hierarchical structure of the MATTS2021 data bank in the form of *an extended tree* can be found in the Supporting Information as an individual file named ‘topology-clustering-extended-tree.png’. It includes details about the number and chemical element types of the first and second neighbors for atom types and information about the group planarity (atom types from 1p, 2p, and 3p groups are marked with bold fonts) and belonging to a three-membered, four-membered, five-membered, six-membered, or seven-membered ring (colored background) according to Figure S3 in the Supporting Information. Clusters at the terminal level of the extended tree show atom types with identical numbers and types of the first and second neighbors.

**Figure 7:**
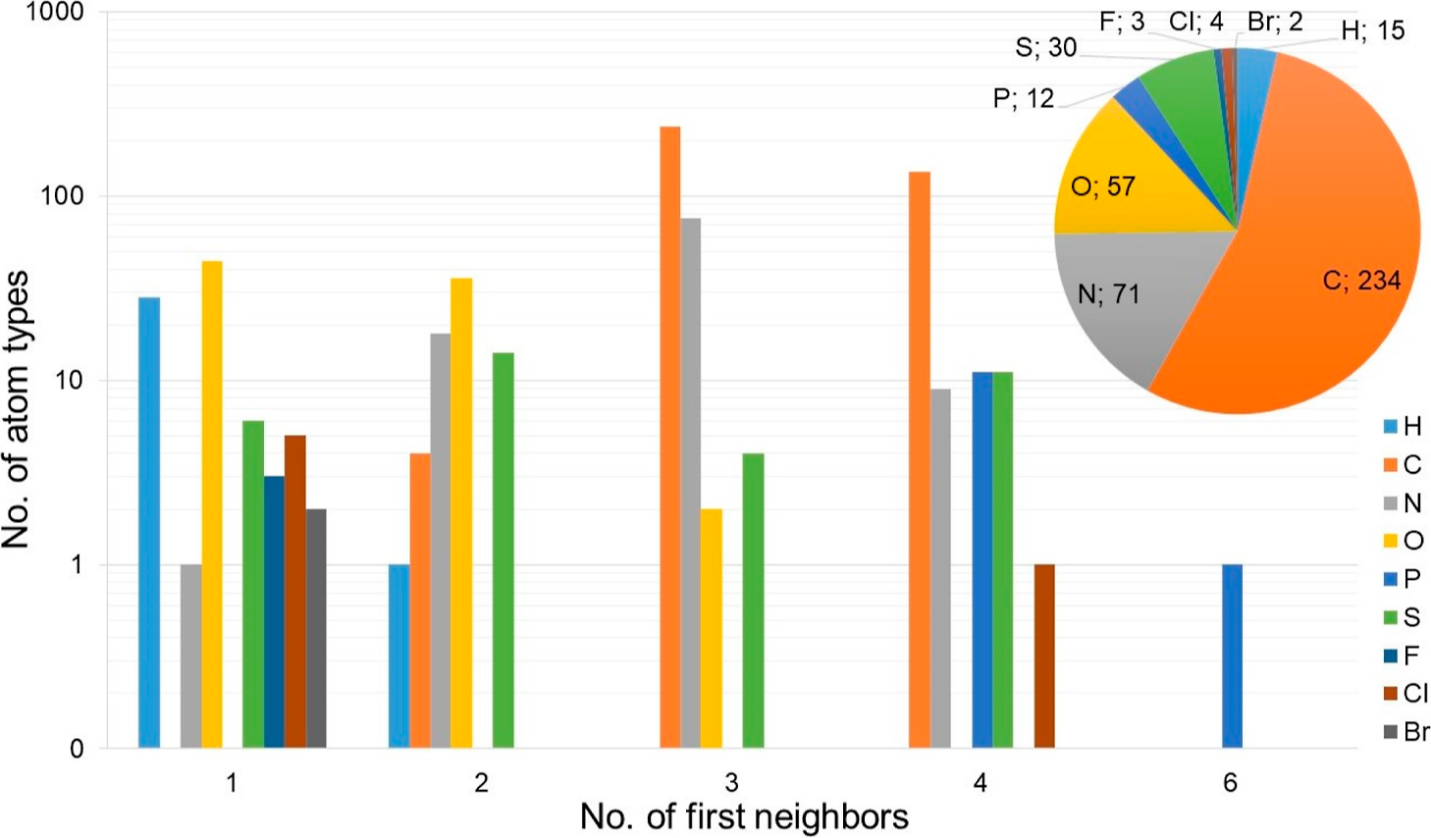
Bar graph of the distribution of atom types with one, two, three, four, or six first neighbors for each chemical element present in the MATTS2021 data bank and a pie chart of the number of clusters created for atom types of each chemical element. The scale on the y axis is logarithmic.

Expanded information about the number of clusters based on their size, chemical elements, and the number of the first neighbors can be found in the Supporting Information in Table S3. The largest, seven-element cluster occurred only once, for C atom types with three first neighbors: C, N, O, and with four second neighbors of any element. The average cluster size is 1.52 atom types. The number of first neighbors varies for atom types of each element. The statistical representation of the MATTS2021 data bank in terms of the two criteria (cluster size and the number of first neighbors) is shown in Figure 7.

Less specific atom types without defined second neighbors are shown higher in the tree structure, and there were 13 of them. Atom types that due to their chemical character cannot have second neighbors, were shown at the terminal level of a tree with both the number and type of second neighbors marked as “none”. There were seven such atom types: N339 (N from NO_3_^–^), N455 (N from NH_4_^+^), O323 (O from H_3_O^+^), O001 (O from H_2_O), P601 (P from PF_6_^–^), S442 (S from SO_4_^2–^), and Cl04 (Cl from ClO_4_^–^)

### 3.2 Results of the Rotation of Local Coordinate Systems

Many new local coordinate systems for atom types were applied during the process of rotation compared to the original ones in the MATTS2021 data bank. Only some of the local coordinate systems used in the data bank remained unchanged. One of criteria for defining an atom type in the MATTS data bank is the sample standard deviation for MM parameters (κ, *P*_val_, κ′, or *P*_*lm*_). In the original MATTS2021 data bank, 11% of atom types has at least one sample standard deviation for *P*_*lm*_ larger than 0.05 e, which was allowed only in the case of either low transferability of electron density expected due to chemical factors or rarity of atom type occurrence.^11^ After rotations, 3298 out of 11 064 data points had at least one sample standard deviation for *P*_*lm*_ larger than 0.05, which makes up 29.81% of the data set. In terms of atom types, 205 of them were marked inconsistent, which makes up 31.5% of the data bank. However, the large percentage of inconsistencies is reasonable as some of the considered rotations would not be optimal. The rest of the parameters (κ, *P*_val_, and κ′) and their sample standard deviations do not depend on the choice of the local coordinate system.

The numbers of entries in the data set increased from 651 atom types (used in the topology clustering) to 11 064 atom types in different local coordinate systems (used in the electron density clustering). Also, due to the different number of possible local coordinate systems for each group (as shown in Table 1), some of the atom types occurred in the data set used for the electron density clustering more frequently than others, especially those from the 4n group.

### 3.3 Density Clustering

**Figure 8:**
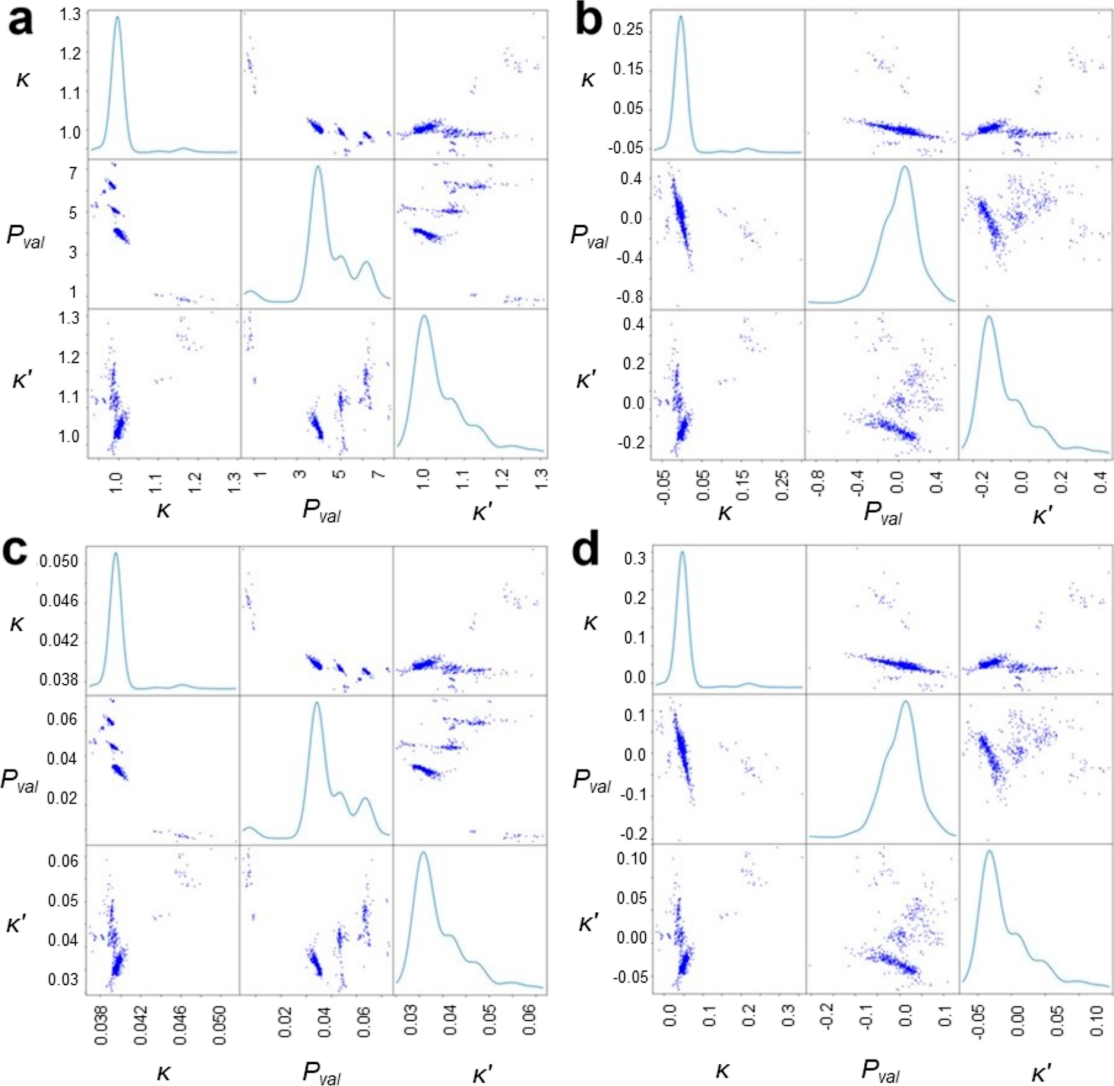
Scatterplot matrices of κ, *P*_val_, and κ′ (a) for original, (b) subtracted, (c) normalized, and (d) subtracted and normalized data.

Figure 8 shows how the data modified by subtraction and/or normalization correspond with the original data. In the case of κ and κ′ parameters, their density plots and a relation plot did not change neither by subtracting nor by normalizing, and only values of the parameters were modified. Contrastingly, for *P*_val_, density plots and correlation plots with κ and κ′ changed significantly after subtracting the number of valence electrons. The dominant effect of κ, κ′, and especially *P*_val_ seen in the original data was reduced most prominently by using both subtraction and normalization. Density clustering was carried out for all three possible modified sets, subtracted data, normalized data, and subtracted normalized data, to compare the clustering results with the original data and to check the usefulness of each approach in general. The subtracted data gave essentially the same results as the original data, both with the number and distribution of clusters, some of which would require the next level of clustering. Using the normalized data resulted in one large cluster, including the majority of the data, and many smaller, usually few-element clusters. The subtracted and normalized data seemed to be the most useful as the number of clusters increased significantly in comparison to clustering on the original data. In each approach, some clusters found in the original set were completely recreated. Considering the above, two ways of electron density interpretation were introduced. First one uses original, unmodified parameters from the MATTS2021 data bank, whereas the second one is based on the subtracted and normalized data, which gives the most similar results to the original data set, and the effects of the dominance of *P*_val_ over the rest of the parameters and the dominance of one or two *P*_*lm*_ within all *P*_*lm*_ values are reduced. With the applicability aspect of the clustering to data bank building process kept in mind and the fact that heavily modified data do not give immediate information about true values of parameters and would be more challenging to interpret, the main focus was on the original data. Full-density clustering results of the original data and their statistical analysis can be found in the Supporting Information as an Excel file named ‘density-clustering.xlsx’.

#### 3.3.1 Impact of Local Coordinate Systems on Multipolar Functions

Introducing a large number of local coordinate systems for atom types had a significant reason–a symmetry higher than that defined in the MATTS data bank can appear in different systems. As shown in Figure 2 in the Methods section, possible local coordinate systems are marked with two colors. Green indicates the minimum set of local coordinate systems required to see all possible symmetries for each group independently. Purple represents the systems necessary to cluster and compare different groups (4n, 3n, 3p, etc.) with one another.

**Figure 9:**
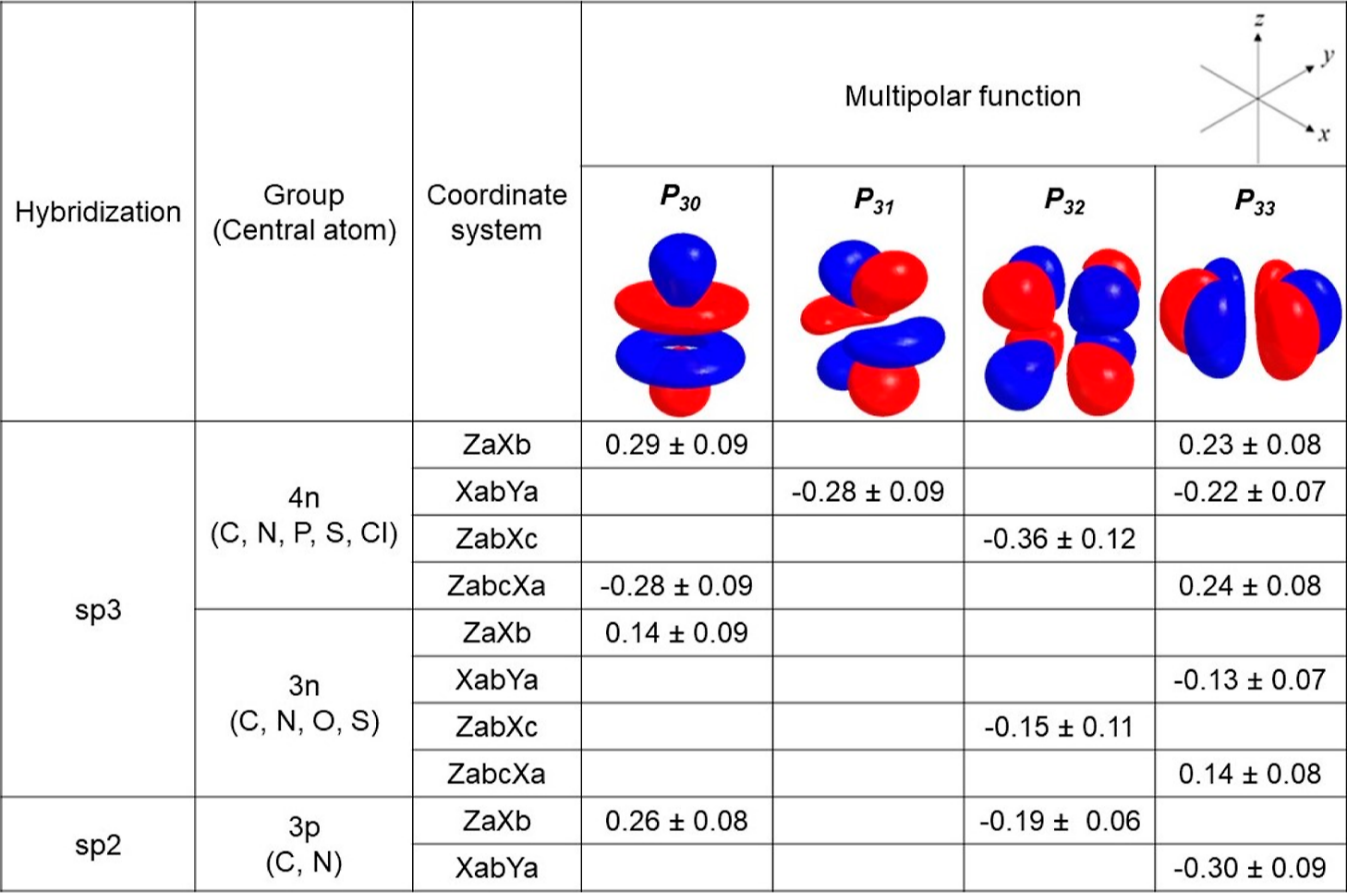
Multipoles with the highest absolute population values for different groups. Values are presented as mean and the sample standard deviation. Visualizations of multipoles are exemplary. The real arrangement of multipoles and more detailed information can be found in the Supporting Information in Figure S4. The effect of multipole domination is not very clear in the remaining groups so they were omitted in the analysis.

Hybridization of the central atom and the choice of local coordinate systems determine which multipoles have the highest absolute population, and this is very well seen in particular clusters (see Figure 9). The numbers in the table were obtained by averaging the *P*_*lm*_ values and show only the dominant ones in each group and local coordinate system. Incorrect values of *P*_32_ for atom types from 3n and 4n groups within the ZabXc system were excluded from the calculations as they were positive instead of negative due to the presence of indistinguishable first neighbors and orienting local coordinate systems on them. Usually only one dominant multipole is present, for example, negative *P*_32_ for the ZabXc system in both 4n and 3n groups, positive *P*_30_ for the ZaXb system and negative *P*_33_ for the ZabcXa system in the 3n group, and positive *P*_33_ for the XabYa system for both 3n and 3p groups. However, when two dominant multipoles are present, their linear combination in correct proportions gives the function of analogous shape as one dominant multipole in the other coordinate system. This occurs not only in the 4n group, with positive *P*_30_ and *P*_33_ for the ZaXb system, negative *P*_31_ and *P*_33_ for the XabYa system, and negative *P*_30_ and positive *P*_33_ for the ZabcXa system, but also in the 3p group for the ZaXb system with positive *P*_30_ and negative *P*_32_. Noteworthy, some multipoles dominant in the 4n group are not present in the 3n group. They were omitted intentionally as the effect of dominance was indiscernible. The reason behind that is probably because the 4n group includes mostly carbon atom types and the 3n group includes nitrogen atom types, which have small *P*_*lm*_ parameters with little variety, so values of the multipoles are determined mostly from them. Additionally, sulfur atom types have higher absolute values of *P*_*lm*_ parameters than other atom types, which also affects the mean and the sample standard deviation of the population of multipolar functions. More meaningful analysis of dominant multipoles and its relation to hybridization can be carried out only if focused on particular clusters collecting similar values of MM parameters, see chapters 3.3.3–3.3.9.

#### 3.3.2 Visualization of Clusters from Density Clustering on General Trees from Topology Clustering

**Figure 10:**
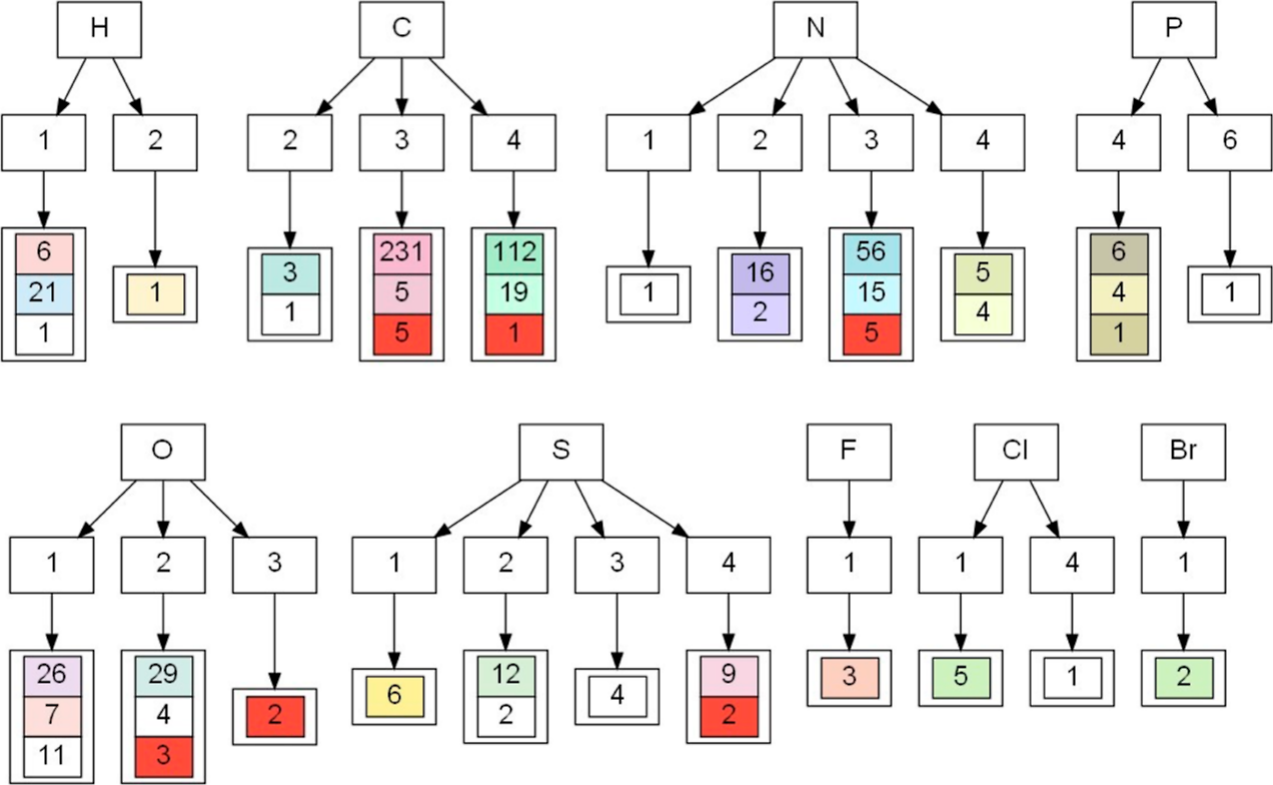
Scheme of clusters created with density clustering, visualized on concise trees, showing the biggest clusters for each combination of the central atom and number of first neighbors present in the data bank. If there are two fields with the same background color but different hues one below the other, the lighter one shows the number of atom types excluded from the main cluster (the one with a darker hue). The red background means that an atom type is “unique”, that is, it undoubtedly differs from other atom types from the same group and creates one-element clusters. The white background implies that atom types are excluded from main clusters and can combine in a more complex way for various reasons.

**Figure 11:**
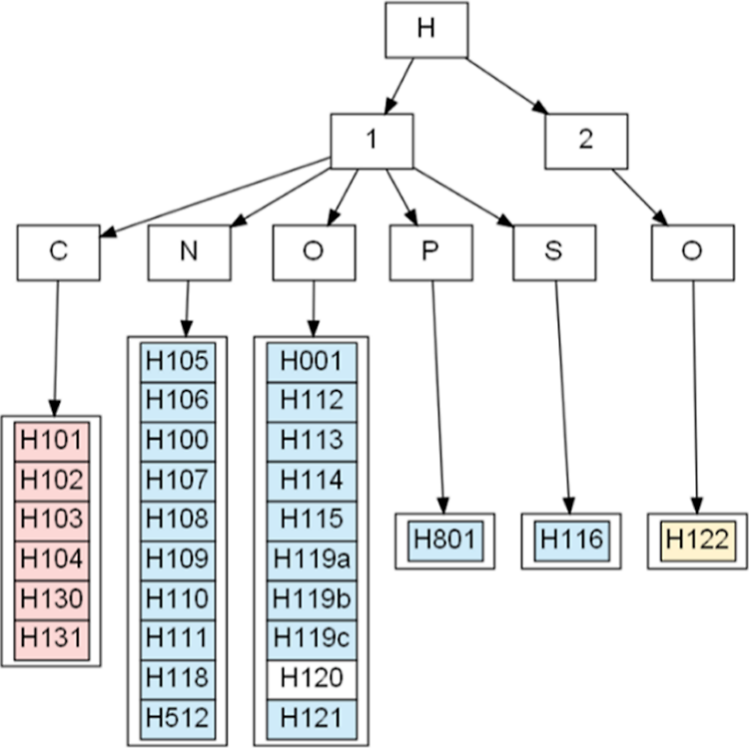
Visualization of density clustering results on a *general tree* for hydrogen atom types.

Topology and density clustering results differ from each other in terms of the size and number of created clusters. From the topology clustering, there were 428 clusters from one to seven atom types. From density clustering, at the final (fourth) level of clustering there were 513 clusters of a size from 2 to 1509 atom types. Also, 270 outliers were found. Apart from the diversity in the distribution of clusters, the outcome of both clustering approaches also varies. Density clustering, using differences in *P*_val_ and *P*_*lm*_ values, maximally separated atom types by the group, chemical element, and rotations, whereas topology clustering focused on separating atom types by the chemical element also but mostly by the number and type of first and second neighbors. To connect topology and density clustering results, topology clustering results were visualized on a *general tree* with information only about the number and element types of first neighbors and density clustering results were incorporated by using a colored background. The overall scheme of how atom types are divided into clusters is shown in Figure 10 in a form of concise trees. The color of the background indicates the biggest, the most general, and universal cluster for each group with labels according to the legend. Noteworthy, due to a large number of possible arrangements of the local coordinate systems for some atom types, especially those from 3n, 3p, and 4n groups, it is challenging to clearly define which cluster is the most representable one, so this division should not be considered as strict but more illustrative. Given the diversity of possible situations in clusters, a few categories of atom types are present. A red background means that an atom type is “unique”, that is, it undoubtedly differs from other atom types from the same group and creates separate one-element clusters. If only one rotation for an atom type was significantly different from others, this data point detached itself completely from other data points during clustering and was labeled with “–1” as an outlier. There are also atom types for which, in some of the local coordinate systems, all or a few of their rotations belong to the ‘large cluster’ and show similarity to other atom types in terms of multipole parameters; however, in other local coordinate systems, they no longer remain in the large cluster and create separate ones. These atom types are named “distinct” and can occur in small clusters individually, in pairs, or in groups and are marked on the extended tree by a lighter hue background. This shows the significance of considering all possible local coordinate systems as in only some of them differences between atom types are visible. A white background implies that atom types are excluded from main clusters and can combine in a more complex way for various reasons explained for each case in the following text. Additionally, information if an atom type belongs to planar (1p, 2p, and 3p) or not planar (1x, 2x, 3n, 4n, and 6n) groups is shown on trees by using bold fonts for planar ones. The actual visualization of density clustering results on general trees is shown in Figure 11 for hydrogen atom types. The rest can be found in the Supporting Information as Figures S5-S11 with the background coloring scheme as explained in Figure S12.

#### 3.3.3 Hydrogen Atom Types

Hydrogen atom types belong in the 1x group with the exception of H122, which is a middle H in the oxonium ion H_2_O_5_^+^ and thus belongs to the 2x group (see Figure 11). Atom types from the 1x group divide into two main clusters (0___ and 1___) in line with expectations from their chemical properties, the separating factor being the type of the first neighbor indicating whether the said hydrogen is polar or nonpolar. In these clusters, *P*_10_ and *P*_20_ multipoles have positive values and the rest are zero as expected (they were never refined in the model molecules). All hydrogen-type clusters exhibit cylindrical symmetry, as expected. More details can be found in the Supporting Information.

#### 3.3.4 Carbon Atom Types

Graphical representation of clustering of carbon atom types, which constitute the majority of the MATTS data bank, is shown in Figures S5 and S6. In the 2x group, there are four carbon atom types; all of them are detached from other carbon-type groups and divided into two categories already at the first level of the clustering. The division has a chemical explanation, because C202, C203a, and C203c are from unsaturated aliphatic hydrocarbons, whereas C201 is a carbon from the nitrile group. All the two carbon 2x clusters exhibit cylindrical symmetry, as expected.

The 3n, 3p, and 4n groups of carbon atom types stay together until the third level of clustering, where the main separation by groups, corresponding to the local coordinate systems available for each group (ZaXb and XabYa for 3p and ZaXb, XabYa, ZabXc, ZabcXa for 3n and 4n), occurs. The 3n group contains only one atom type—C3a2, which is almost planar and seems similar to types from the 3p group in all local coordinate systems common for these two groups. In groups 3p and 4n, large clusters for each coordinate system are created, and they contain the majority of carbon atom types that for given conditions of the analysis (Eps, levels of clustering, sample standard deviation etc.) are similar. Additionally, at each level of clustering starting from the second one, unique carbon atom types are observed.

For the carbon 4n group, containing 132 atom types, the situation is similar to the carbon 3p group. In each of the four local coordinate system types possible, a large cluster with majority of the same atom types is present. Distinct atom types are present in clusters for all of the coordinate systems. The sample standard deviations of MM parameters of the largest clusters of the carbon 4n group usually do not exceed the desired values or exceed them only slightly. However, the 4n carbon group is the one which has also large percentage of atom types labeled with the inconsistence flag.

In all the clusters, *P*_*lm*_ parameters obey the maximum symmetry possible for a particular coordinate system and agree with the sp^3^ shape of electron density. Similarly like with the 3p group, the XabYa coordinate system allows the largest cluster of similar atom types to form. The most restrictive is the ZabXc system, suggesting that only 68 atom types for which all rotations belong to the 10_0_16_3 cluster truly have 4̅3m symmetry. There is only one unique atom type which does not appear in any of the above large clusters, and it is C840. More details can be found in the Supporting Information.

#### 3.3.5 Nitrogen Atom Types

Nitrogen atom types may belong to 2p, 3n, 3p, or 4n groups; there is also one atom type in the 1x group: N101. The 2p group, containing 18 atom types, with 16 of them having C and N atoms as second neighbors. All the clusters show the highest possible symmetry, and sample standard deviations of their parameters do not exceed the desired values. It seems that it would be possible to define a general atom type for the nitrogen 2p group.

Approximately 70% of 3p nitrogen atom types cluster together with each other in clusters 9_4__ (ZaXb system) and 9_5__ (XabYa system). Each of the clusters also contain the majority of 3n nitrogen atom types. Both clusters contain the same atom types; they only differ because of different rotation types. Coexistence of atom types from two groups, 3n and 3p, in one cluster shows the significance of using ‘purple’ local coordinate systems (shown previously in Figure 2) designed to allow such situation.

sp^2^ hybridization for nitrogen types from the 3p group follows chemical intuition; however, it is somehow unexpected to see that majority of nitrogen atom types from the 3n group also seem to have electron density resembling more sp^2^ than sp^3^ hybridization. Deeper investigations are required to better understand possible causes of this observation (specific geometry, not unique enough definition of coordinate system, etc.). Clusters 9_7__ and 9_8__ containing only 3n nitrogen types have *P*_*lm*_ values which do not fully follow sp^3^ hybridization. In the ZabXc system, the *P*_32_ has the largest absolute value but the *P*_30_ has not disappeared, whereas in the ZabcXa system, the *P*_33_ is not balanced well by the *P*_30_, the latter being ca. twice smaller. This is most probably because electron density of the lone electron pair at nitrogen atoms has to be solely described by nitrogen atom multipole functions, whereas electron density of covalent bonds is usually described by multipolar functions of both, the central nitrogen atom and its covalent neighbors. Thus, multipolar functions contributing to lone electron pair descriptions have to have higher populations.

The nitrogen 4n group creates clusters based on the local coordinate system. Within each cluster, MM parameters have their sample standard deviations smaller than the desired threshold, whereas their mean values follow clearly the electron density shape of the sp^3^-hybridized atoms and fulfill requirements for the highest possible symmetries to be seen in a particular coordinate system. More details can be found in the Supporting Information.

#### 3.3.6 Oxygen Atom Types

Oxygen atom types belong to the 1p, 2p, or 3n group and cluster in a complicated way, which can be seen in Figure S8. None of the oxygen atom types received label of being inconsistent. 44 atom types from the 1p group are divided into two main clusters that differ from each other in terms of the *P*_22_ and *P*_val_ parameters.

Co-clustering of 1p and 2p oxygen types from two different coordinate systems (ZaXb and ZabYa) can be understood while looking at pictures visualizing local coordinate systems (see Figures 3 or S1), remembering that 2p is derived from 4n with lone electron pairs in place of atoms c and d and 1p is derived from 3p with lone electron pairs in place of atoms b and c. Such a combination of the number of first neighbors and local coordinate system orientation allows to orient the system the same way with respect to lone electron pairs. Existence of the mixed clusters suggest that these oxygen types are not contributing any electron density to bonding with its covalent neighbors, but they do contribute to lone electron pair density. Nevertheless, departure of oxygen types from asphericity is relatively small, *P*_*lm*_ values are much smaller than those observed for carbon types.

Sample standard deviations for all MM parameters for all large clusters of oxygen types are below the threshold values. The mean values of the *P*_*lm*_ parameters in the case of cluster 2_0_3_1 and 2_3_0_ points toward sp^3^ hybridization of 2p and 3n oxygen types belonging to them; however, here dipole and quadrupole functions have larger populations than octupoles, opposite to what was observed for carbon clusters. Again, departure from an ideal set of populations typical for the sp^3^ hybridization results from the need to describe the lone electron pairs. More details can be found in the Supporting Information.

#### 3.3.7 Phosphorus Atom Types

Phosphorus atom types belong either to the 4n or 6n group, the latter including only one atom type, P601, which naturally is unique. The remaining 11 types are highly divided (do not form any large cluster but a few smaller ones). It might be because, in general, *P*_*lm*_ values of phosphorous types are among the highest observed for all atom types and the *Eps* value optimal for the entire data set is already too small for phosphorus types to keep them together. More details can be found in the Supporting Information.

#### 3.3.8 Sulfur Atom Types

Visualization of density clustering results on a general tree for sulfur atom types is shown in Figure S10. All six sulfur atom types from the 1p group create one cluster (4___). Sample standard deviations of MM parameters for the cluster are small and acceptable. From the mean *P*_*lm*_ values, it is clear that the types do not have cylindrical symmetry the *P*_22_ has not averaged to zero, although it has a smaller absolute value than *P*_20_ and *P*_30_.

The situation is more complicated for the remaining groups of the sulfur atom types. In the 2p group, most atom types are grouped together—one cluster for each local coordinate system (ZaXb, XabYa, and ZabYa). Sample standard deviations of parameters are acceptable. Symmetries resulting from the *P*_*lm*_ values are the highest possible to be easily spotted within each of the coordinate system. Electron densities of these types fulfill the sp^3^ hybridization, although electron density lobes originating from sulfur atoms and directed to the covalent partner are different from those directed to the positions of the two lone electron pairs.

Next, there is the 3n group that contains only four sulfur atom types, and three of them (S320, S399, and S001) cluster together in three of four possible local coordinate systems, ZaXb, XabYa, and ZabcXa, but separate in the ZabXc system. Regarding symmetries resulting from the *P*_*lm*_ values, the situation is strange. For the ZabcXa system, the symmetry is much lower than the maximal possible, m instead of 3m, although the multipoles violating 3m symmetry have smaller populations than the one fulfilling it. For the XabYa system, the symmetry is too high. Apparently, the system focuses too much on only two neighboring atoms, not describing properly contributions from the third neighbor and lone electron pair.

Finally, in the 4n group with 11 atom types, there are two clear unique types, that is, S411 and S442. The four largest clusters of sulfur 4n types have acceptable values of sample standard deviations of their parameters, and sulfur 2p and 3n clusters almost follow the requirement for the sp^3^ hybridization. Here, although no lone electron pairs are expected, all the four lobes are directed to chemical bonds. Apparently, the bonds are not equivalent and the observed symmetry is *mm*2 (not 4̅3*m* nor 3*m*), although again the multipoles violating the symmetry have much smaller absolute populations with respect to those the most populated. In fact, for sulfur atom types, multipoles dominant in each group have significantly larger absolute values than the rest of the atom types, with exception for phosphorous types which have the highest. More details can be found in the Supporting Information.

#### 3.3.9 Chlorine, Bromine, and Fluorine Atom Types

The least numerous atom types present in the MATTS2021 data bank are halogens: only three F, two Br, and six Cl. All of them belong to the 1p group, except for the Cl04 atom type that corresponds to the chlorine atom in the perchlorate ClO_4_^–^ ion and thus belongs to the 4n group and differs from other chlorine atom types. All fluorine atom types cluster together (30___), as shown in Figure S11. Similarly, bromine and chlorine atom types cluster together, which suggests their chemical similarity (5___). All the halogen clusters have low sample standard deviations for density parameters, and all of them exhibit cylindrical symmetry. More details can be found in the Supporting Information.

#### 3.3.10 Density Clustering of Data Modified by Subtraction and Normalization

As mentioned previously, there was a second clustering approach considered, using not the original data from the MATTS data bank, but modified by subtraction and normalization. In this case, the effect of some parameters dominating over others is noticeably reduced. The most valuable advantage of using the subtracted and normalized data is a significant increase in the number of clusters created already at the first level of clustering (1037 clusters instead of 92), which include atom types in a way corresponding to results from the clustering using original data. However, such division happened for manually selected *Eps* = 100, significantly smaller than the one found by the knee method (*Eps* = 455.859). The Eps parameter found using the algorithm divided the data set only into two clusters: H atom types and the rest. The gradual decrease in *Eps* resulted in the appearance of more clusters. Noteworthy, such large values of *Eps* were due to the multiplication of the data set with 11 064, which did not change the distribution of data in any way but was used only to simplify the analysis as the differences between parameters after subtraction and multiplication were small. Large clusters present in the clustering with original data are in this case greatly divided to the point where much smaller groups can be easily observed. With the overwhelming number of clusters, there is no possibility to discuss all of them here but to give some examples: carbon atom types, previously mostly in a few large clusters, now create many small groups such as {C303a, C303b, C378a, C382b, C382d, C383b, C510, C590, C777, C780}, {C3890, C3891, C523, C784, C888}, {C374a, C514, C568b, C813}, {C953a, C953b}, {C779, C837, C837b, C838b, C838c}, {C330, C332, C3a3}, {C313a, C313b, C3133, C3134, C3134, C702}, {C318, C516a, C516b, C516e, C516f, C521s}, {C329c, C959}, {C311, C316c}, {C309b, C310, C321}, {C300, C312, C304, C308, C328}, or {C301, C238}. Generally, there is no need for further division after the initial clustering due to the fact that the most suitable *Eps* was chosen manually; however, in some cases, especially for carbon atom types, given that they make the majority of the MATTS data bank, the next levels of clustering for smaller *Eps* make even more detailed and adequate groups.

## 4 Discussion

### 4.1 Main Factors that Decide about the Electron Density Distribution of Atom Types

The atom types from the MATTS2021 data bank were divided into clusters by DBSCAN of MM parameters, up to four levels of clustering. Results from each level gave its own insight into how clustering progressed, why atom types created clusters in the way that they did, and led to establishing factors affecting the division of clusters on each level. It turns out that only a few factors decide about similarities and dissimilarities between electron densities of atom types and are the reason for dividing the main, large cluster. At the first level, atom types were separated by the chemical element of central atom, except for two cases. Chlorine and bromine types stayed together, as well as oxygen and 2p sulfur types. Thus, the first and most important factor in differentiating electron densities of atom types is the chemical element of the central atom, which corresponds with *P*_val_. However, a part of clusters obtained at the first level had sample standard deviations for mean *P*_*lm*_values larger than 0.05 e. For this reason, the second level was performed for clusters with carbon, nitrogen, oxygen, and sulfur types, and third and fourth levels were performed for carbon and oxygen types. At the fourth level of clustering, clusters contained atom types of only one chemical element, group, and local coordinate system, with a few exceptions, such as cluster of atom types with chlorine and bromine atoms, mentioned above. Also, 1p oxygen types with the ZaXx system and 2p oxygen types with the ZabYa system stayed together. Another reasonable exception is the chemical similarity between different groups, like 3n and 3p nitrogen atom types, which did not separate during the clustering. Thus, we concluded that the number of first neighbors and planarity of the group are the second most important factors during the clustering. Impact of these factors on relationships between atom types is revealed only after the first level of clustering. The number of first neighbors and planarity of the group directly translate to the type of rotation.

Another descriptor of atom types, the ring membership to the planar ring, was not a clear factor differentiating between the clusters. On one hand, many carbon atom types from 3p and 4n groups that belong to three- or four-membered rings turned out to be distinct. On the other hand, in the large clusters that should gather similar atom types, there were types with various ring memberships, making it inconclusive to clearly state the importance of this factor during the clustering.

The symmetry of the atom types does not seem to be the dividing factor at any level of clustering, so these atom types must have the highest possible symmetry or their departure from the highest possible symmetry is very small. The average image of the group is almost always consistent with the highest symmetry—calculating average values of *P*_*lm*_ parameters for the largest clusters allows us to assign symmetry according to Table S2, which is presented in Figure S12.

Among other factors that influence the electron density of an atom type, there are a few that need to be addressed. Their role as factors distinguishing atom types was not clearly stated as they were not directly used as atom types’ descriptors in the clustering but can be concluded from other available information. Regarding first neighbors, their variety of chemical element types, presence of lone electron pairs, the number of lone electron pairs, and the number of such atoms among the first neighbors seem to be important. This conclusion arises from the topology characteristic of unique and distinct atom types, especially carbon types, where a lot of them have two or more first neighbors other than carbon and hydrogen. The division between atom types with carbons and hydrogen as first neighbors versus other chemical elements is certainly seen for hydrogen types (polar and non-polar) and 2x carbon types.

### 4.2 About the Unique and Distinct Atom Types

The large clusters formed by the clustering show that there are many atom types described in detail that seem to be similar and perhaps can be redefined into more general ones. Combining the atom types into more general types can increase the recognizability of atoms in molecules using the MATTS data bank. The first level of electron density clustering reveals the occurrence of unique atom types that detach from the other atom types and create small separate clusters. Therefore, it will not always be possible to create a general type resulting from similarities in both MM parameters and topology between atom types. Some atom types refer to very specific chemical groups with a unique electron density distribution. Thus, they have to be described precisely.

Distinct atom types have a special characteristic—they appear in the large cluster in one of the local coordinate systems and can no longer be present in respective large clusters in other local coordinate systems. The answer for such a behavior is quite straightforward—some local coordinate systems reveal features that differentiate distinct atom types from others. The question lies in *what* are those features. In most cases, the number of first neighbors, planarity of the group, and ring membership are enough to see why an atom type shows dissimilarity to other types. However, in some cases, it is uncertain why this particular atom type is distinct.

The clustering results on one hand justify the need for multiplicity of atom types that are distinguishable, when one universal definition is impossible to create. For example, distinct and unique atom types have to be preserved in the data bank. On the other hand, some clusters include many atom types with similar electron density and topology descriptors, which could be included into more general types. Currently, no general atom types are present in the MATTS data bank. It is possible that our set of descriptors used to characterize atom types is incomplete, and new descriptors should be added to facilitate the process of differentiating between general and distinct/unique atom types.

### 4.3 Clustering Shows Three Main Chemically Interpretable Sets of Atom Types

Atom types present in the MATTS data bank may be divided into three sets, based on how they behave in the presence of covalently bonded neighbors: hydrogen types, carbon types, and heteroatom types. The first set, hydrogen types, was divided by clustering into two subsets—aliphatic hydrogen types and polar hydrogen types. The similarity of hydrogen types inside these subsets suggests a possibility of introducing more general definitions for some of them. Secondly, carbon types are sensitive to their neighbors and adapt to them. It seems that four levels of clustering are insufficient, and many more levels would be necessary to divide the created clusters. Thirdly, there are heteroatom types such as oxygen, nitrogen, sulfur, phosphorus, and halogen types. They divide rather quickly during the clustering and contribute less to bonds than to electron pairs. Oxygen types respond to their neighbors poorly, focusing on describing their own lone electron pairs. Because of this, they cluster together and are indistinguishable. This implies that the definitions of oxygen types in the data bank were too detailed. Nitrogen types are something between carbon and oxygen types, and they adjust to the neighbors, unless a lone electron pair is present. Sulfur types are highly divided already at the first level of clustering based on their multipole populations, and maxima and minima of deformation electron density are strengthened compared to carbon atom types. Due to the small variation of phosphorus types, hardly any conclusion can be derived about them. Despite a small number of halogen atom types, it is visible that chlorine and bromine types bonded to one neighbor are very similar and their MM parameters cluster together.

### 4.4 Some Local Coordinate Systems are More Preferable than Others

The process of rotation created some local coordinate systems that were universal and some more fitted for the group that atom types belonged to. Thus, local coordinate systems treated neighboring atoms in an unbalanced way. Some of them, like ZabXc or ZabcXa, engaged three neighbors (a, b, and c), whereas others, like ZaXb, XabYa, or ZabYa, engaged only two neighbors (a, b), leaving out the third one. The least restrictive systems, XabYa and ZabYa, engaged only two neighbors placing the first axis in the average direction between them, not accenting any of the two neighbors. Next, the ZaXb system focused on one of the neighbors, placing the *Z*-axis in its direction. Finally, the most restrictive systems, ZabXc and ZabcXa, engaged three neighbors. For the 4n group, the best local coordinate system would engage all four neighbors. We do not currently have a suitable software to perform this task, but it is possible, and one such local coordinate system had appeared in the literature before.^33^ From such variety of available local coordinate systems arises the question that how to find the most optimal system, that is, in which system, the proper symmetry will be shown. If an atom type has the highest symmetry and equal neighboring atoms, it will always create the same clusters independently from the type of coordinate systems and combination of neighbors used to define the system. However, there are situations when different local coordinate systems give different clusters, so neighboring atoms are unequal and the symmetry of an atom type is lower than the highest possible. The presence of distinct atom types indicates the inequality of neighbors and that only some rotations are proper. Future research should focus on finding a useful way of determining which local coordinate systems describe the proper symmetry of atom types but some conclusions can be made already. The XabYa system can be used for the 3p group, even though it engages only two neighbors. This is because adding a third neighbor to the coordinate system is impossible in planar groups. Contrastingly, the XabYa system should not be used in non-planar groups as it is not differentiating enough. Also, there is a possibility that by using not restrictive enough local coordinate systems, the symmetry can be overestimated. Then, checking if neighbors are distinguishable or not would show which systems are the most appropriate.

On the other hand, rotation with inconsistent parameters occurred for many atom types. The problem of inconsistency arises from averaging atom types with unequal neighbors, so averaging something that cannot be considered as the same. The number of atom types with inconsistent parameters varies between atom types of different chemical elements and groups but is especially high among the 4n carbon group. Inconsistencies make it challenging to fully interpret the results as it is not clear to what extent they influence the change of electron density parameters and their possible error.

### 4.5 Many Dimensions, Many Difficulties

Having data in 18 dimensions makes the analysis a challenging task. Some aspects of problems typical for multidimensional analysis occurred, such as troubles with defining the *Eps* parameter in DBSCAN. Because some of the data points were extremely close to one another, whereas other were scattered around, the knee method for finding the optimal *Eps* was not working correctly. The value found using the algorithm for the whole data set did not discriminate between the points that were close to one another and was the reason why four levels of clustering were introduced. Nonetheless, the knee method made the analysis easier, omitting the step of “guessing” the *Eps* value. There were also some contradictions between algorithmically determined clusters and clusters that would be reasonable from a chemical point of view. This situation happened for the same reason as discussed earlier—*Eps* was not dividing the clusters with sufficient accuracy and next levels of clustering were needed (as shown in Table 2), where different groups or local coordinate systems were mixed. The approach using the subtracted and normalized data with manually chosen *Eps* could be a promising tool to find atom types that are similar in a less time-consuming way because some levels of clustering are omitted. Still, the data were heavily modified, which should be kept in mind during future work and interpretation of clustering with this approach.

Ideally, we would like to have an algorithm that automatically creates clusters with certainty that atom types within a cluster are similar enough for making a justified definition of a general atom type and shows which characteristics are responsible for the resemblance. Finally, visualizing multidimensional data is troublesome; however, to some extent, we were able to determine distances between atom types from interpreting values of their parameters.

### 4.6 Machine Learning as a New Approach to Get Electron Density

With the increasing significance of using computational methods in chemistry, many approaches to obtaining electron density based on machine learning (ML) have been introduced through the years. In majority, they implement DFT and neural networks to construct electron density. There are a few properties of a good ML model: transferability between various systems, ability to learn from small data sets, and accuracy in predicting electron density from high- and low-density regions. Obtaining electron density using ML is less time-consuming than directly with DFT. However, with the increasing number of heavy atoms in the molecule, the computational cost of calculations also increases. Currently, the literature shows ML algorithms for molecules with around eight–nine heavy atoms.^45,46^ These methods are not yet suitable for macromolecules as they would produce time and memory problems. Another issue of obtaining electron density with ML methods using quantum mechanics calculations is the accuracy of results that depends on the combination of the input data set of molecules for model training, basis sets used for calculations, and the type of implemented ML method. Generating electron density by using experimental geometry and building atom types has some advantages over ML with theoretical approaches. In ML techniques, the quality of the description would be proportional to the frequency of occurrence. Thus, it would be dominated by the most frequently found atoms. In our approach, we have a larger control of how well the exotic types, obtained from rare chemical connections in molecules, would be described. Secondly, when defining atom types, their definitions can be expanded using the chemical knowledge to further increase accuracy. The data bank approach allows us to understand the relations existing between electron densities of atoms, so wise selection of model molecules is crucial at the beginning stage. Applying chemical knowledge can minimize the number of model molecules needed to include in the data set. Without this knowledge, a much larger set of model molecules would be necessary. However, to fully describe atom types, expanding a set of model molecules is inevitable and using ML methods would be reasonable to obtain new atom types and construct future versions of the MATTS data bank.

### 4.7 Future Outlook

Considering many local coordinate systems for each atom type may reveal a more optimal local coordinate system than the currently used one in the MATTS data bank. Such analysis has not been performed yet. We expect that it will make the data bank more consistent and ordered without changing the types’ definitions. Perhaps, clustering performed not on atom types, but on atoms, would get around the problems of distinguishing neighbors and determining preferable local coordinate systems, but we currently are not able to establish a simple solution for this. Ideally, the most optimal system would be found from the clustering by the analysis of multipole parameters in different systems, without enforcing symmetry. Maybe optimization within a group of atom types, not the entire data bank, could be successful. Additionally, introducing atom types with more accurate and broad definitions would be a significant upgrade as it could cover a wider range of atoms and allow for better recognition. We would like to use ML in the next stage of the project; thus, the deep understanding of the data is necessary beforehand. This work and its results should be considered as an initial stage.

## 5 Conclusions

Information on electron density for all atom types in the MATTS data bank was analyzed for different orientations of the local coordinate systems. Clustering based on the MM parameters describing the electron density was carried out, and the obtained clusters were analyzed in terms of similarities and differences. Results were then compared with groups from clustering by topology, arranged in a hierarchical tree structure. Despite the two different approaches for choosing factors categorizing the data bank into groups, results largely overlap with each other. Moreover, it turns out that the element of the central atom, the number of first neighbors, and planarity properties of the group are three factors that differentiate MM parameters of atom types the most. Those descriptors can be used to introduce the general atom types with less strict definitions. The influence of other descriptors reveals the distinct/unique atom types while most of the remaining types stay the same. The existence of distinct and unique atom types is the main obstacle in creating general atom types’ definitions covering the entire chemical space. Possibly, finding new descriptors would solve the problem of generating more accurate but still general definitions of atom types.

Future development of the data bank will lead to achieving the eventual goal of having all atom types necessary to model crystal structures of all known molecules. Thus, a new way of atom typing based on a statistical analysis of similarities and dissimilarities of electron density parameters would be desired. Nonetheless, the currently available version of the transformed data bank has sufficient accuracy for fast modeling and reconstructing the electron density of many important molecules.

## Supporting Information Available

Computational details and expanded information about electron density clustering results (PDF)
Excel file with all the obtained results and the one used for DBSCAN (XLSX)
Image of an extended tree that shows topology data in the form of a hierarchical structure (PNG)
Compressed folder with bash and Python scripts (ZIP)

## Supplementary Material

ci2c00145_si_002.xlsx

ci2c00145_si_003.png

## Notes

Data used and analyzed during this study are provided in the Supporting Information in the ‘density-clustering.xlsx’ file. Local programs and scripts necessary to reproduce the results published in this paper are available in the Supporting Information. The current version of the data bank, MATTS2021, can be printed out from one of the DiSCaMB application programs available for download free of charge for non-profit users from the webpage http://4xeden.uw.edu.pl/software.
