# Supplementary material for: Multipolar Atom Types from Theory and Statistical Clustering (MATTS) Data Bank: Impact of Surrounding Atoms on Electron Density from Cluster Analysis
Source: J Chem Inf Model. 2022 Aug 9;62(16):3766–83. doi: 10.1021/acs.jcim.2c00145 (PMC9400106; doi:10.1021/acs.jcim.2c00145)
Supplement: Supplementary file 1 — ci2c00145_si_001.pdf [file ci2c00145_si_001.pdf]

Supporting Information

Multipolar Atom Types from Theory and Statistical Clustering (MATTS) Data Bank: Impact of Surrounding Atoms on Electron Density from Cluster Analysis

Paulina Maria Rybicka, Marta Kulik, Michał Leszek Chodkiewicz, Paulina Maria Dominiak**

Biological and Chemical Research Centre, Department of Chemistry, University of Warsaw, ul. Żwirki i Wigury 101, 02-089, Warszawa, Poland

*Correspondence e-mail: m.kulik@uw.edu.pl, pdomin@chem.uw.edu.pl

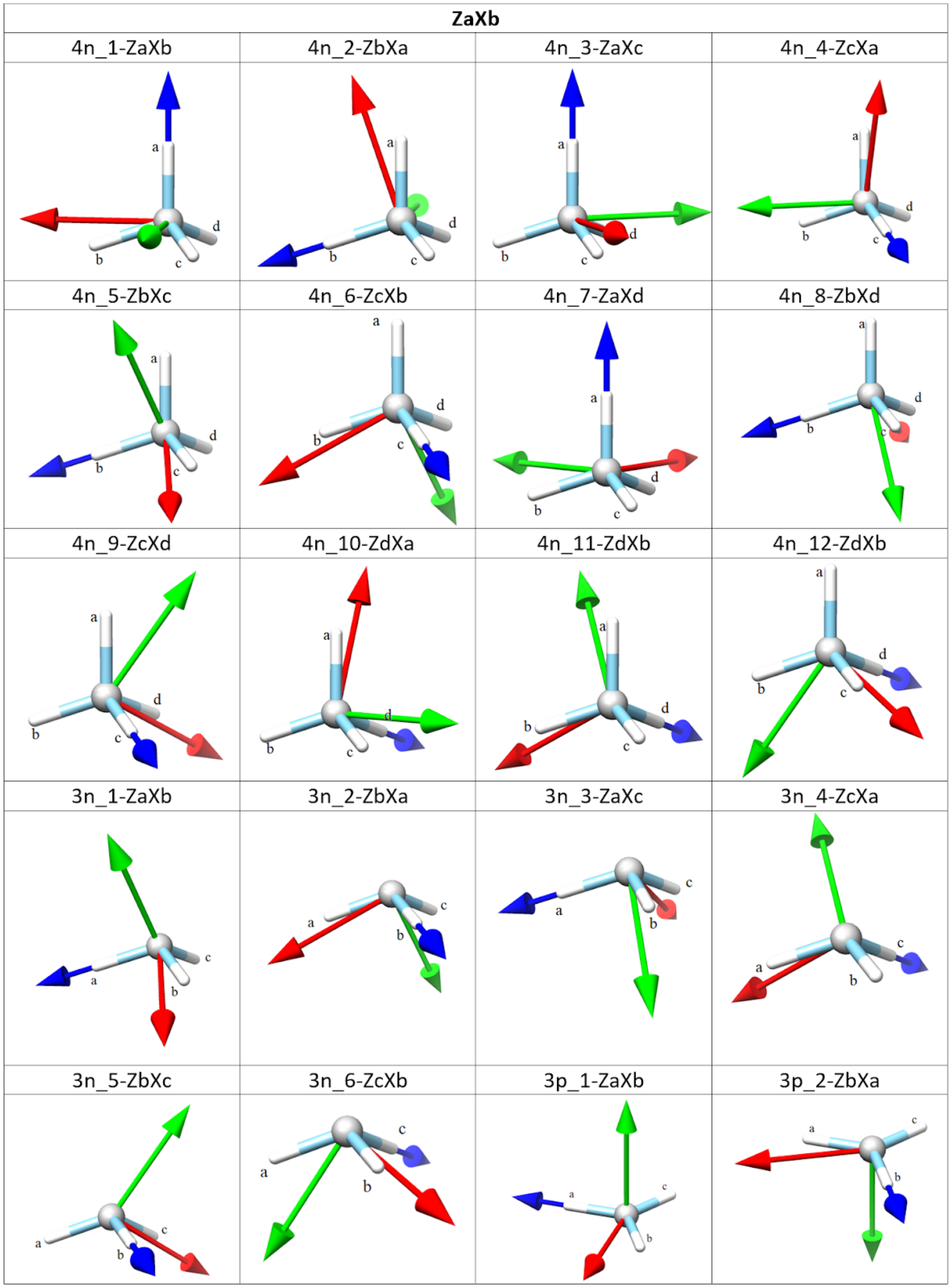

Figure S1 (part 1). All rotations of local coordinate systems.

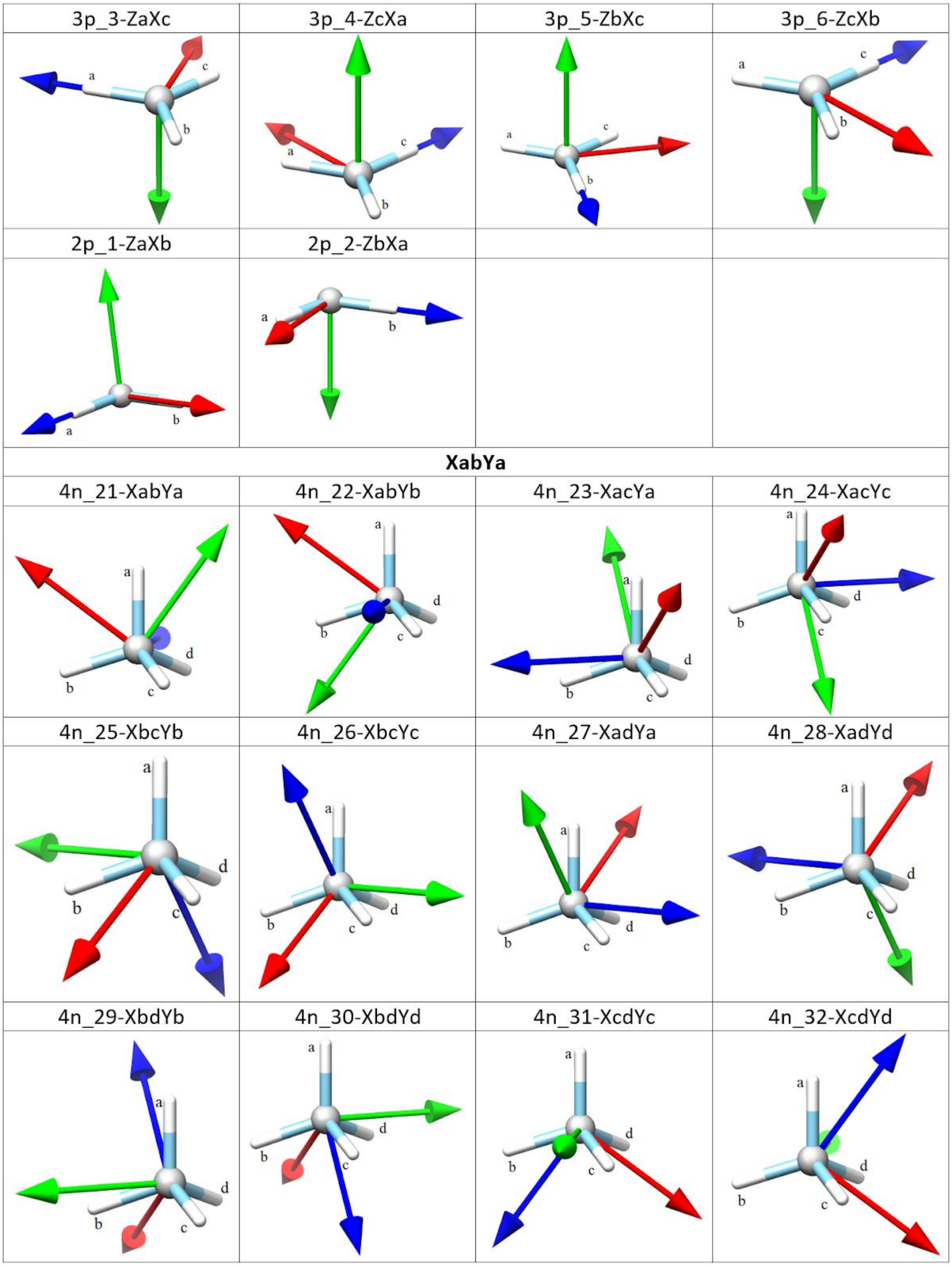

Figure S1 (part 2). All rotations of local coordinate systems.

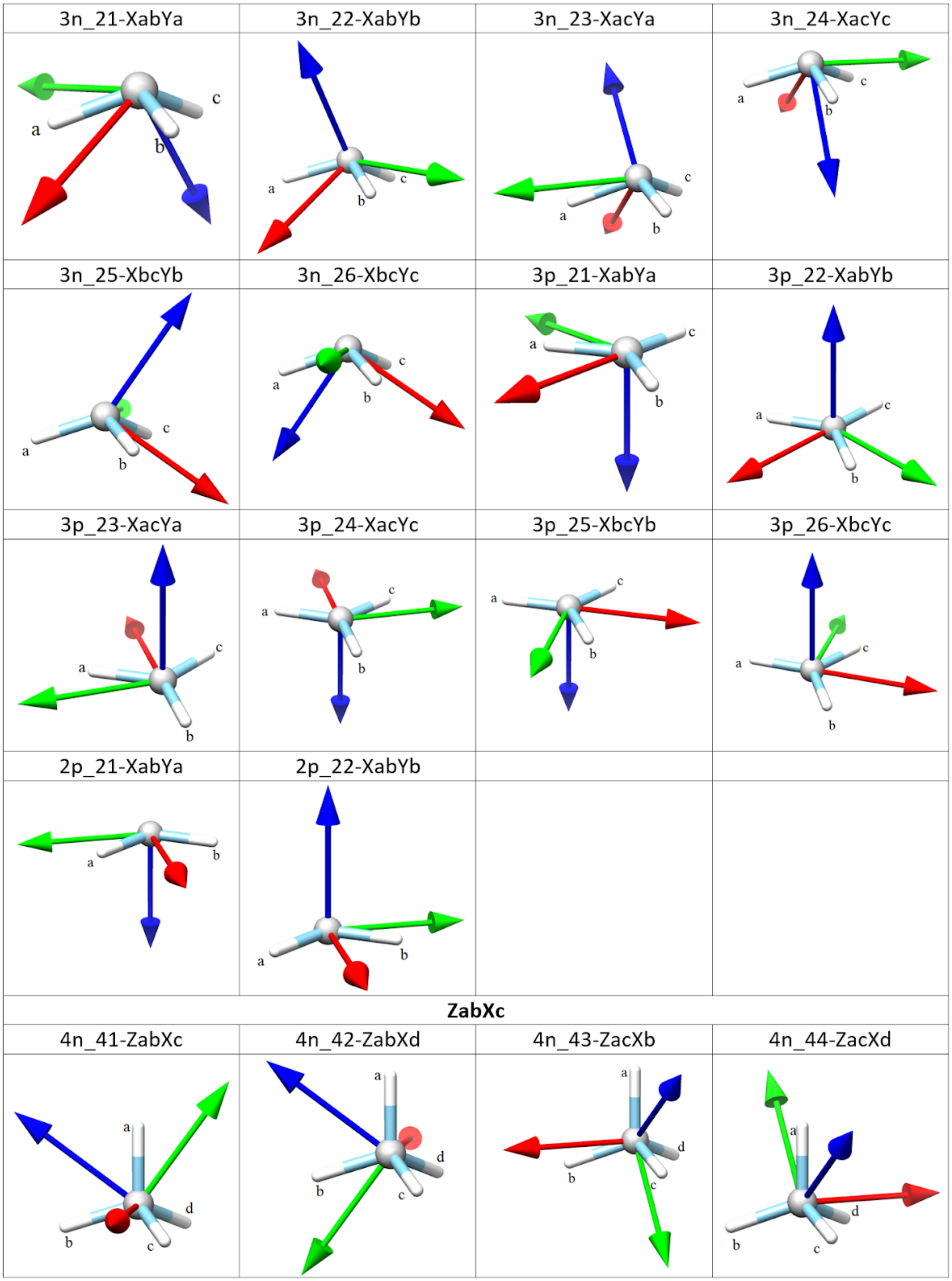

Figure S1 (part 3). All rotations of local coordinate systems.

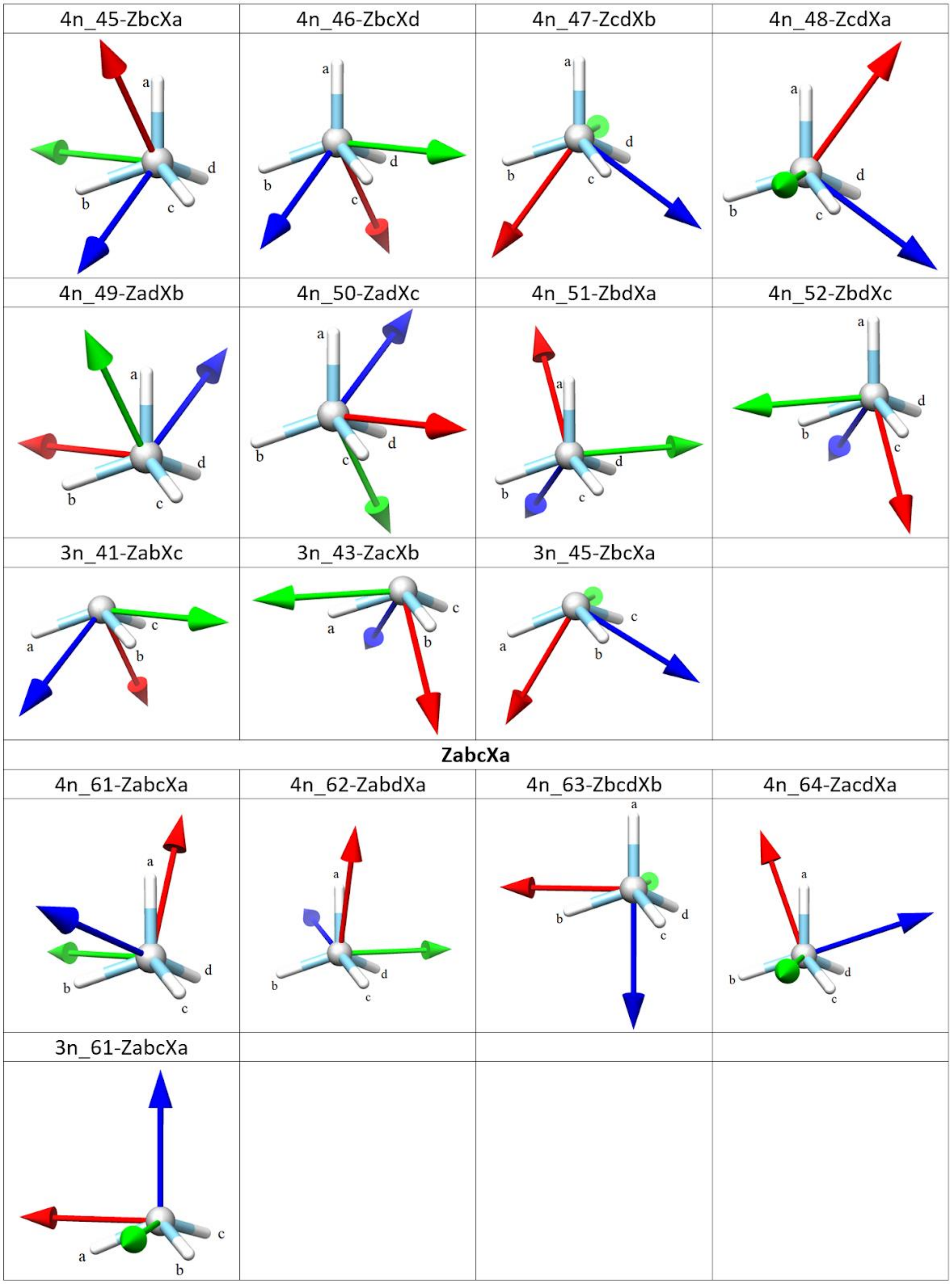

Figure S1 (part 4). All rotations of local coordinate systems.

ZabYa			
2p_71-ZabYa	2p_71-ZabYb		
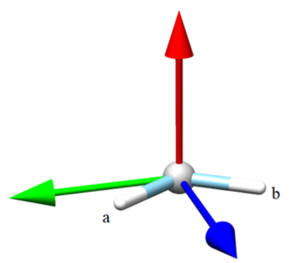	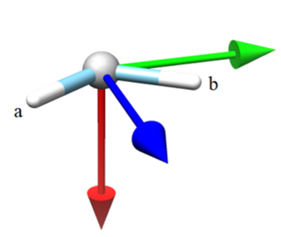		

Figure S1 (part 5). All rotations of local coordinate systems.

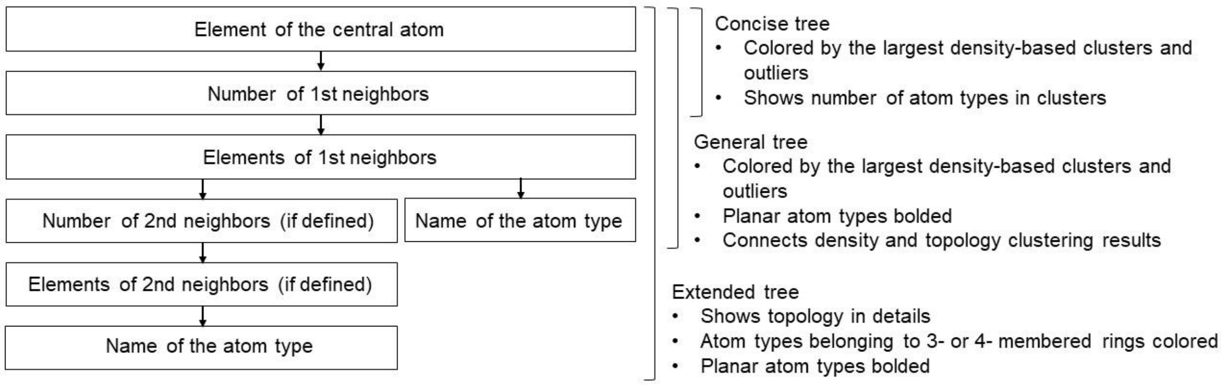

Figure S2. A scheme of types of trees defined for the analysis.

Background color	Planarity	In 5-, 6-, or/and 7-membered rings	In 3-membered ring	In 4-membered ring
**C001**	+ or *	+	*	*
**C001**			-	-
**C001**		-	-	+
**C001**			-	-
C001	-	-	+	*
C001			-	+
C001			-	-

Figure S3. Color scheme used for describing planarity in an extended tree. Symbols: + atom belongs to a ring, - atom does not belong to a ring, * ring membership is not defined. Bolded font indicates that an atom type belongs to a planar group.

Hybridization	Group (Central atoms)	Coordinate system (Rotation numbers)	Multipolar function			
			P_{30}	P_{31}	P_{32}	P_{33}
sp3	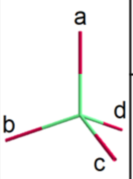 4n (C, N, P, S, Cl)	ZaXb (1-12)	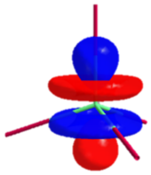			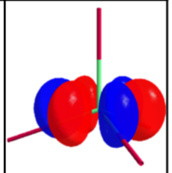
		XabYa (21-32)		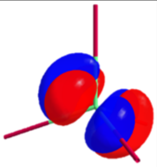		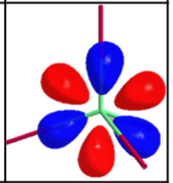
		ZabXc (41-52)			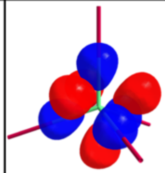	
		ZabcXa (61-64)	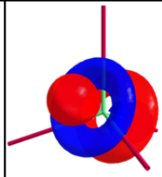			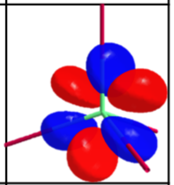
	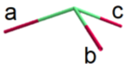 3n (C, N, O, S)	ZaXb (1-6)	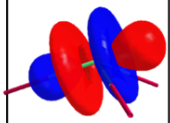			
		XabYa (21-26)				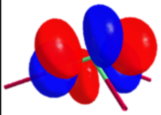
		ZabXc (41,43,45)			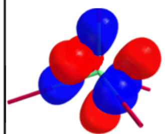	
		ZabcXa (61)				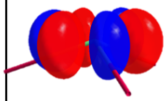

Figure S4 (part 1). Multipoles dominant in each local coordinate system for the most representable groups. Blue – positive, red – negative surface values.

<table>
<tr><td rowspan="2">sp2</td><td rowspan="2">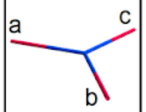
3p
(C, N)</td><td>ZaXb
(1-6)</td><td>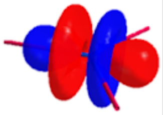</td><td></td><td>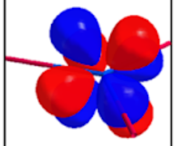</td><td></td></tr>
<tr><td>XabYa
(21-26)</td><td></td><td></td><td></td><td>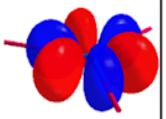</td></tr>
</table>

Figure S4 (part 2). Multipoles dominant in each local coordinate system for the most representable groups. Blue – positive, red – negative surface values.

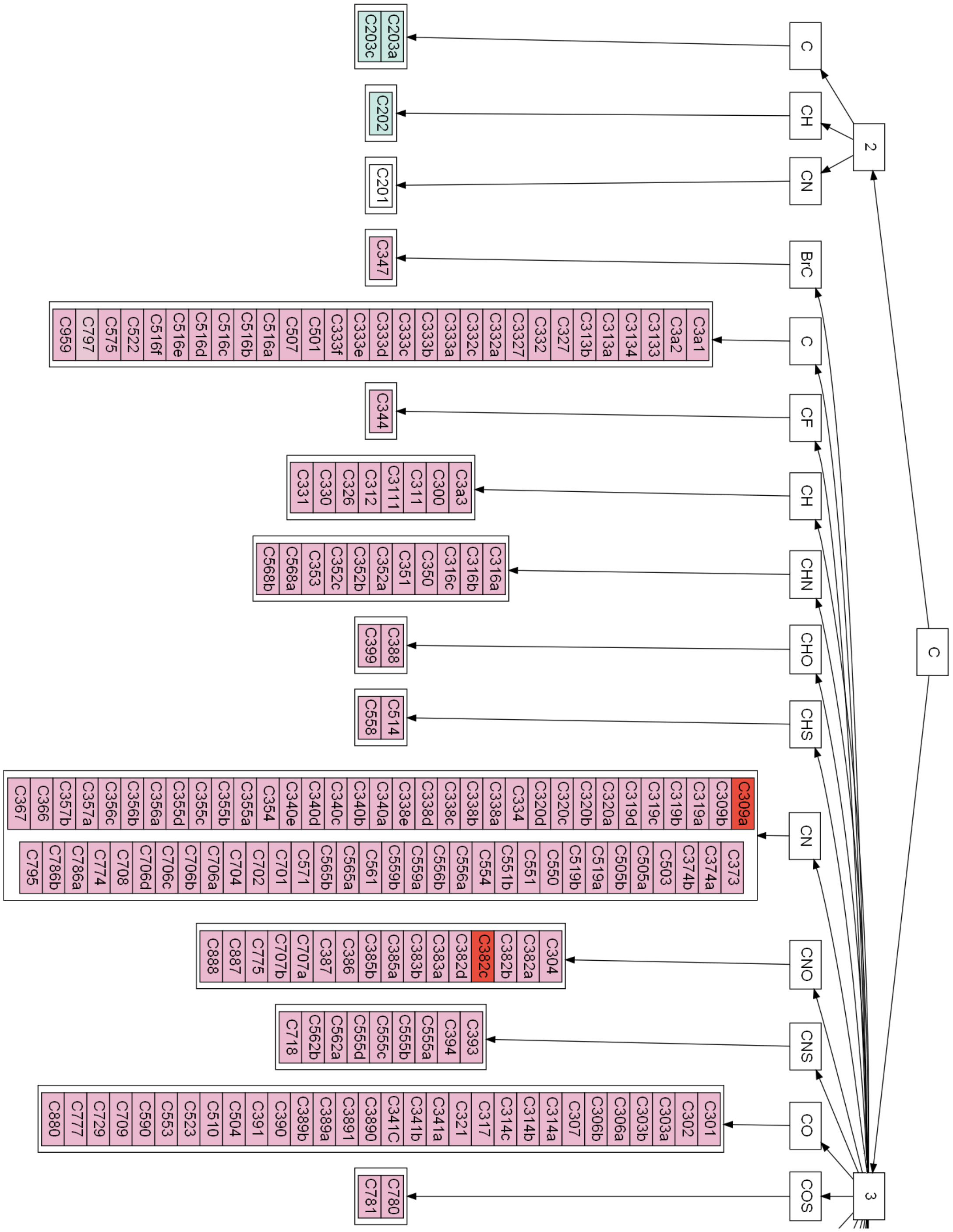

Figure S5 (part 1). Visualization of density clustering results on a general tree for carbon atom types with two and three neighbors. Red – unique atom types, white – specific way of clustering. Bold font indicates that an atom type is planar. Legend for colored background and cluster labels can be found in Figure S12.

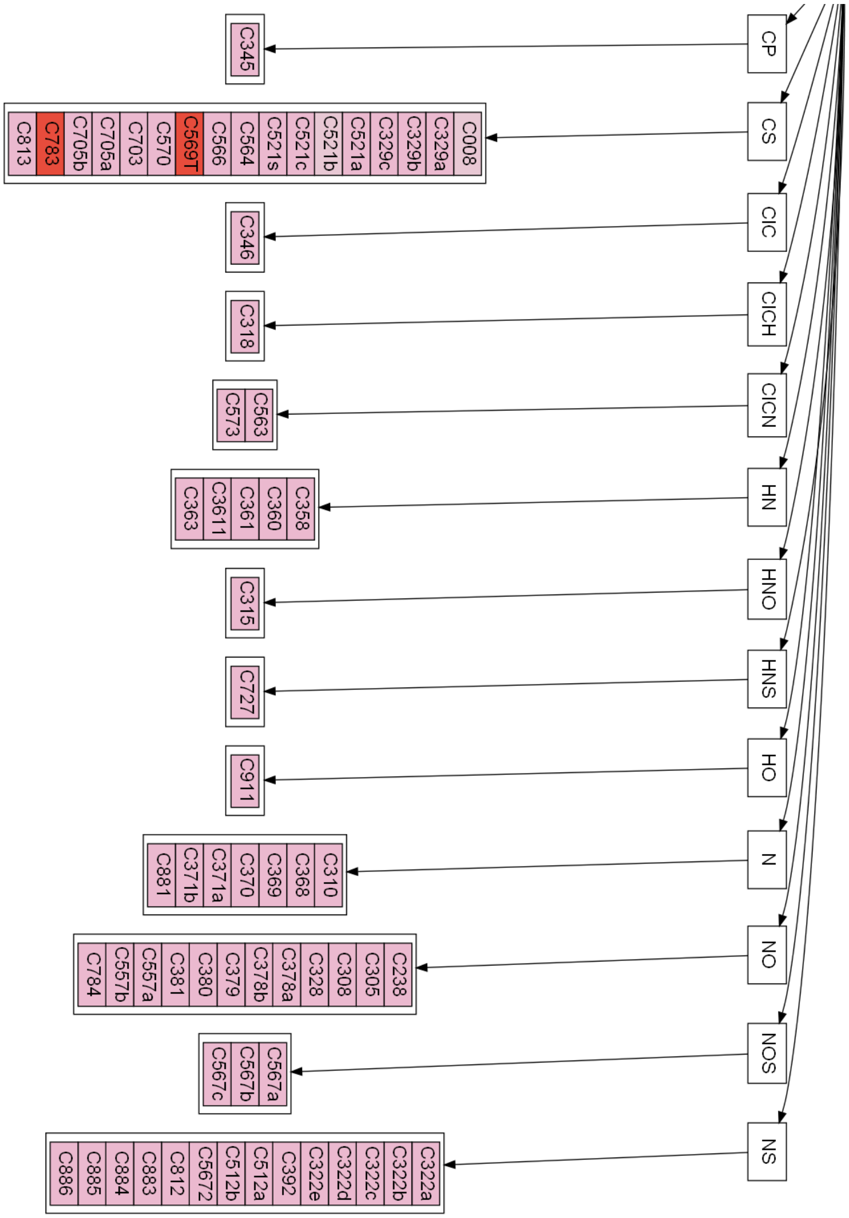

Figure S5 (part 2). Visualization of density clustering results on a general tree for carbon atom types with two and three neighbors. Red – unique atom types, white – specific way of clustering. Bold font indicates that an atom type is planar. Legend for colored background and cluster labels can be found in Figure S12.

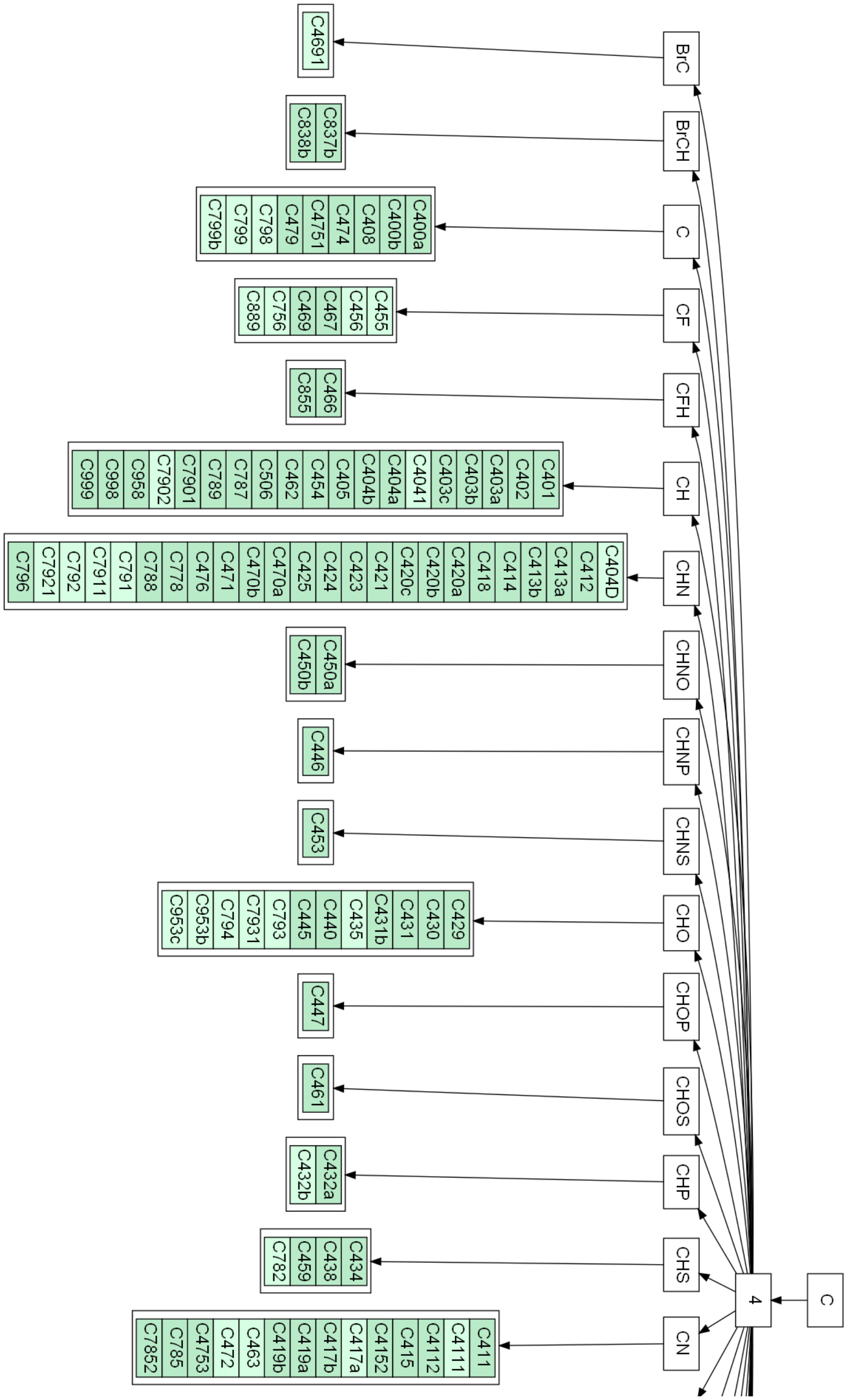

Figure S6 (part 1). Visualization of density clustering results on a general tree for carbon atom types with four neighbors. For the description of details please see caption of Figure S5.

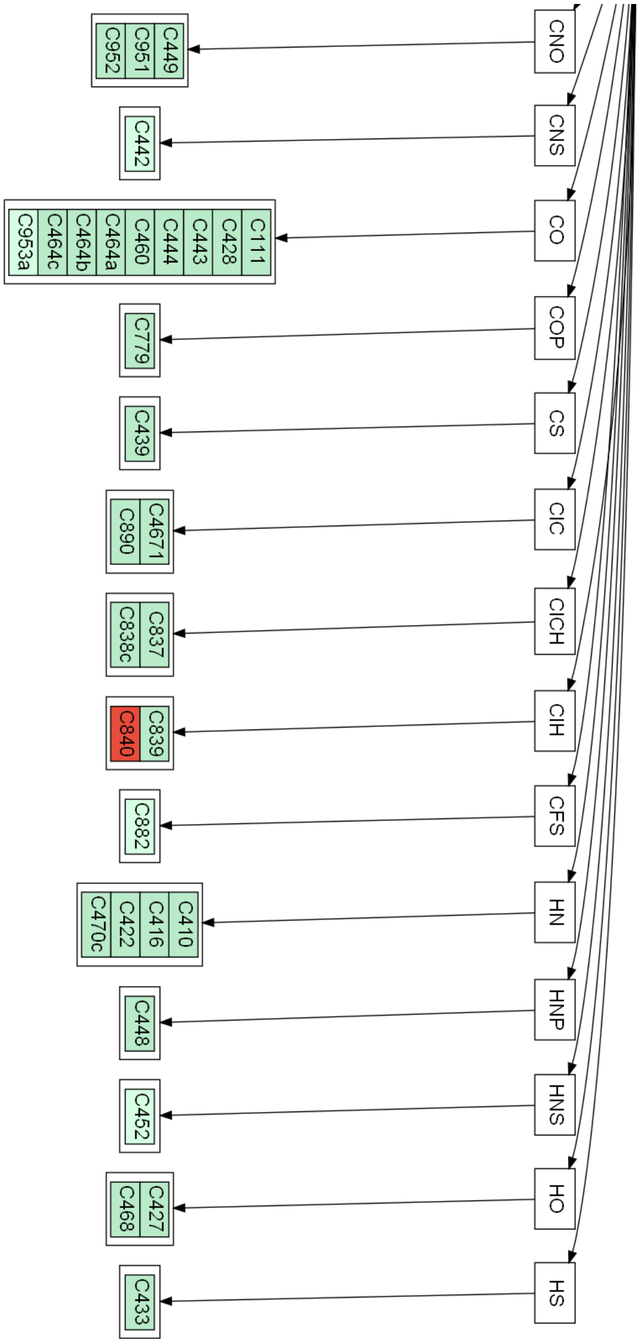

Figure S6 (part 2). Visualization of density clustering results on a general tree for carbon atom types with four neighbors. For the description of details please see caption of Figure S5.

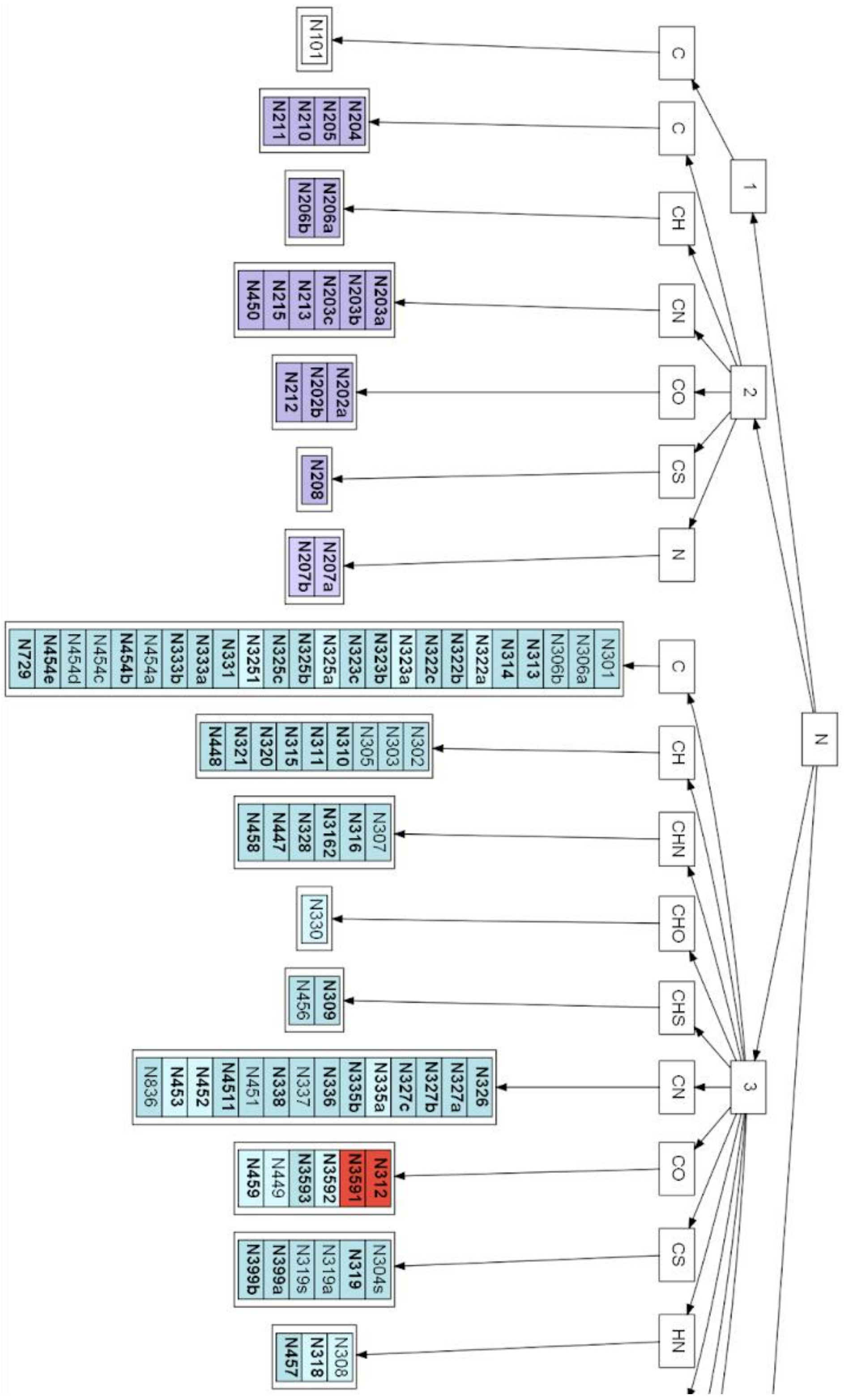

Figure S7 (part 1). Visualization of density clustering results on a general tree for nitrogen atom types. For the description of details please see caption of Figure S5.

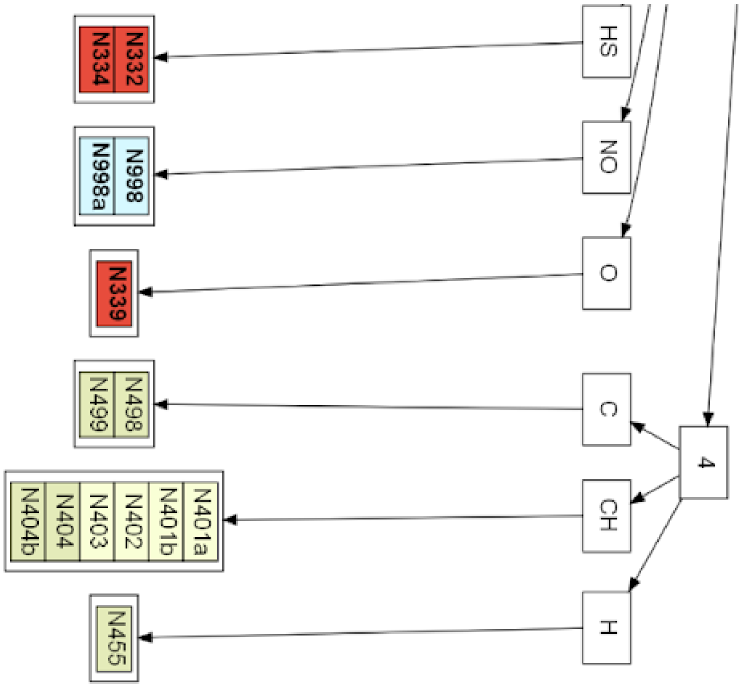

Figure S7 (part 2). Visualization of density clustering results on a general tree for nitrogen atom types. For the description of details please see caption of Figure S5.

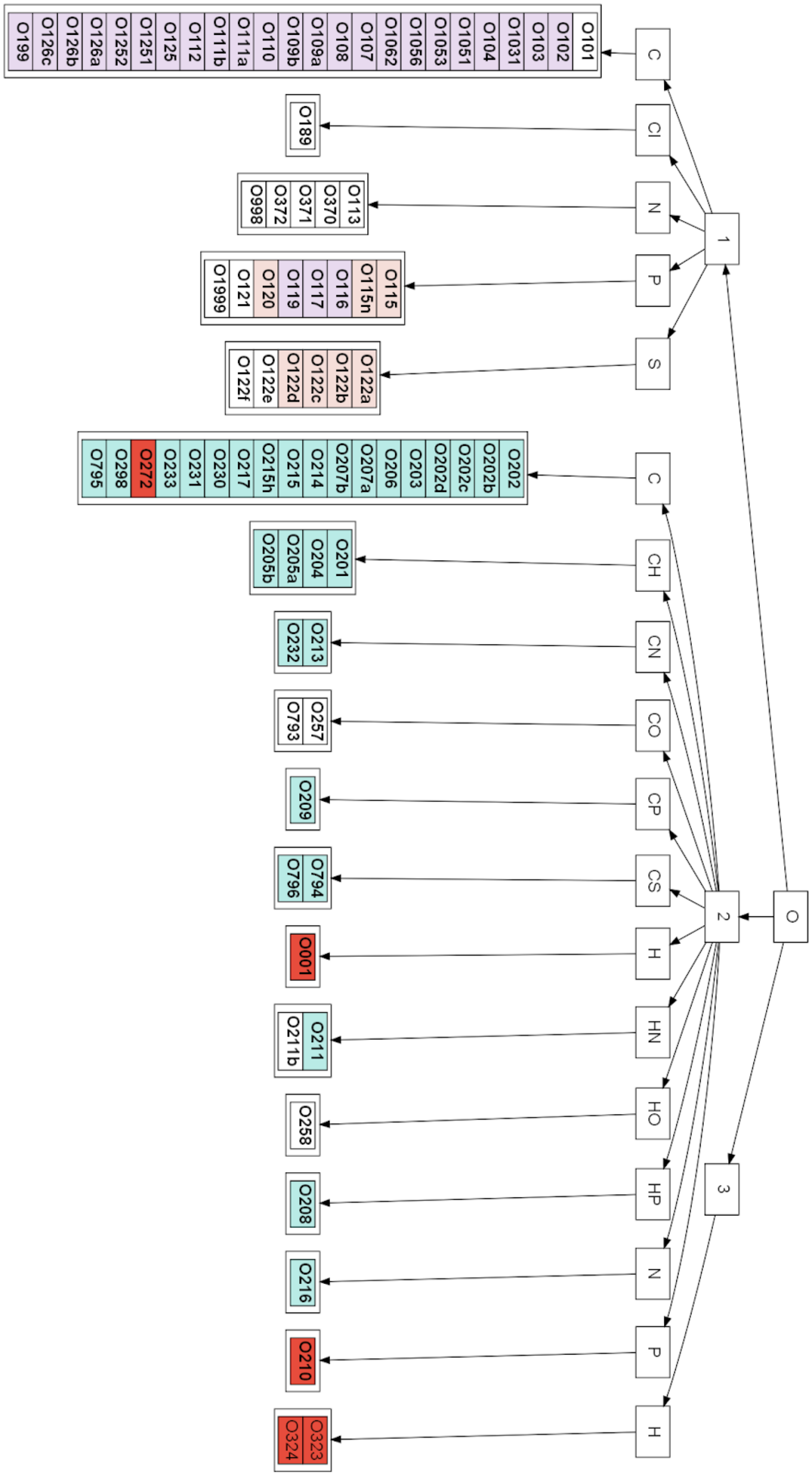

Figure S8. Visualization of density clustering results on a general tree for oxygen atom types. For the description of details please see caption of Figure S5.

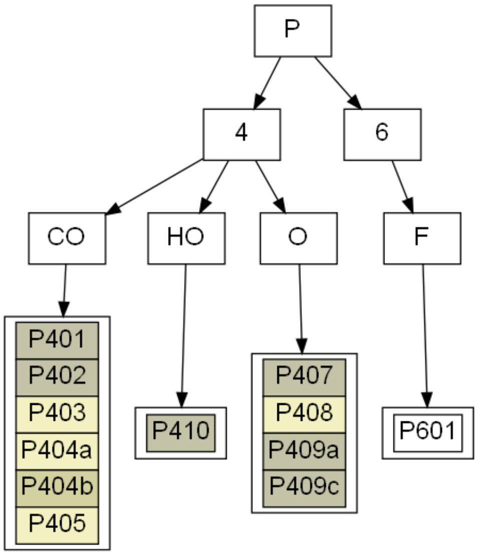

Figure S9. Visualization of density clustering results on a general tree for phosphorus atom types. For the description of details please see caption of Figure S5.

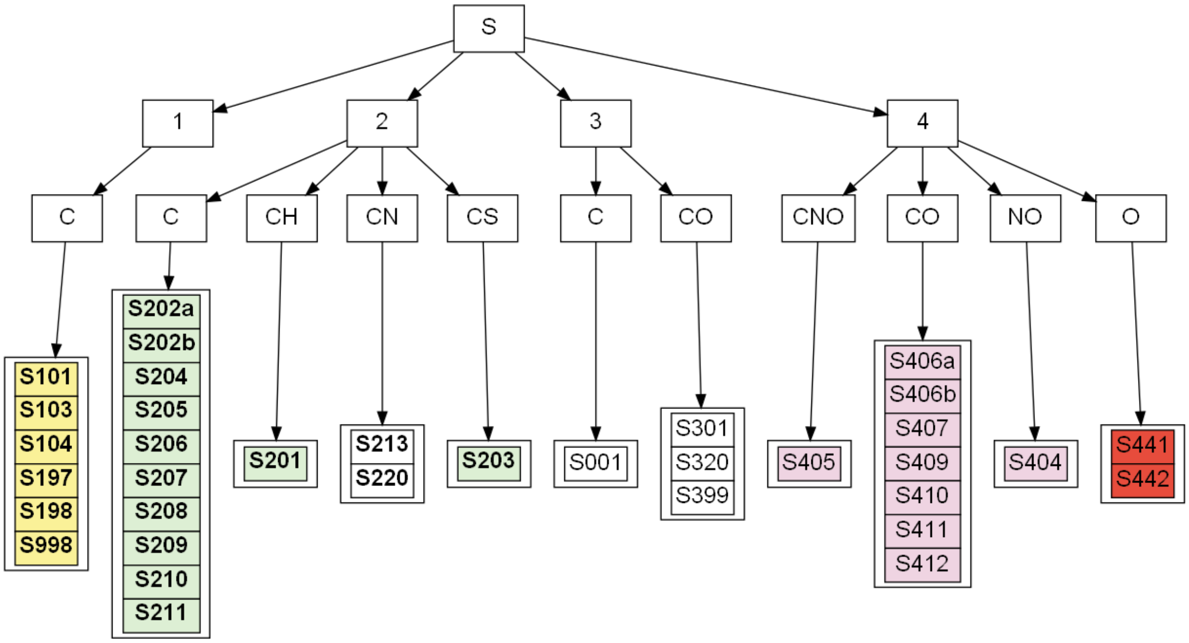

Figure S10. Visualization of density clustering results on a general tree for sulfur atom types. For the description of details please see caption of Figure S5.

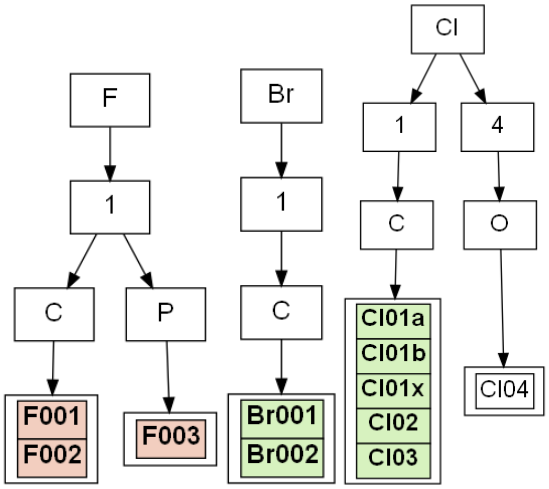

Figure S11. Visualization of density clustering results on general trees for fluorine, bromine and chlorine atom types. For the description of details please see caption of Figure S5.

Element of an atom type	Group	Number of all atom types	Local coordinate system	Background color	Cluster label	Number of atom types with all rotations in the cluster	Number of atom types with at least one rotation in the cluster	Symmetry from the mean parameters	Mean value																	
									κ	P_{val}	κ'	P_{1-1}	P_{10}	P_{11}	P_{2-2}	P_{2-1}	P_{20}	P_{21}	P_{22}	P_{3-3}	P_{3-2}	P_{3-1}	P_{30}	P_{31}	P_{32}	P_{33}
H	1x	28	ZaXb (ZaXany_orthogonal)		0___	6	6	cyl	1.11 ± 0.01	1.06 ± 0.03	1.15 ± 0.01	0	0.19 ± 0.03	0	0	0	0.09 ± 0.01	0	0	0	0	0	0	0	0	0
					1___	21	21	cyl	1.17 ± 0.02	0.85 ± 0.07	1.39 ± 0.07	0	0.14 ± 0.03	0	0	0	0.06 ± 0.01	0	0	0	0	0	0	0	0	0
	2x	1	ZaXb (ZaXany_orthogonal, ZbXany_orthogonal)		6___	1	1	cyl	1.15 ± 0.00	0.59 ± 0.00	1.39 ± 0.00	0	0	0	0	0	0.01 ± 0.00	0	0	0	0	0	0	0	0	0
C	2x	4	ZaXb		7___	3	3	cyl	1.00 ± 0.00	4.04 ± 0.00	0.84 ± 0.00	0	0.16 ± 0.06	0	0	0	0.33 ± 0.02	0	0	0	0	0	-0.09 ± 0.02	0	0	0
					8___	3 (the same as above)	3	cyl	1.00 ± 0.00	4.04 ± 0.00	0.84 ± 0.00	0	-0.16 ± 0.06	0	0	0	0.33 ± 0.02	0	0	0	0	0	0.09 ± 0.02	0	0	0
	3n, 3p	1, 241	ZaXb		10_0_0_0	191 (3p) + 1 (3n)	228 (3p)+ 1 (3n)	mm2 (2||z)	1.00 ± 0.01	4.04 ± 0.09	0.87 ± 0.00	0	0.00 ± 0.07	0.00 ± 0.07	0	0	0.10 ± 0.07	0.00 ± 0.07	0.15 ± 0.06	0	0	0	0.28 ± 0.06	0.00 ± 0.05	-0.21 ± 0.05	0.00 ± 0.03
			XabYa		10_1_0_0	235 (3p) + 1 (3n)	236 (3p) + 1 (3n)	-6m2 (m_|_y)	1.00 ± 0.01	4.04 ± 0.10	0.88 ± 0.03	0	0	0.00 ± 0.07	0.00 ± 0.07	0	-0.19 ± 0.07	0	0.00 ± 0.07	0.00 ± 0.03	0	0.00 ± 0.02	0	0.00 ± 0.02	0	-0.33 ± 0.07
	3n	1	ZabXc		11___	1		m (m_|_y)	1.00 ± 0.00	4.05 ± 0.00	0.88 ± 0.00	0.00 ± 0.02	0	0	0	0	0.06 ± 0.00	-0.06 ± 0.05	-0.12 ± 0.01	0	0	0.00 ± 0.02	-0.21 ± 0.02	0.09 ± 0.01	-0.19 ± 0.01	0.02 ± 0.01
			ZabcXa		-1___	1																				
	4n	132	ZaXb		10_0_16_2	84	117	3m (m_|_y)	1.01 ± 0.01	3.84 ± 0.09	0.92 ± 0.02	0.00 ± 0.01	0.00 ± 0.03	0.00 ± 0.03	0.00 ± 0.02	0.00 ± 0.01	0.00 ± 0.06	0.00 ± 0.03	0.00 ± 0.04	0	0	0	0.26 ± 0.03	0.00 ± 0.01	0.00 ± 0.02	0.21 ± 0.04
			XabYa		10_1_0_1	119	131	mm2 (m||x)	1.01 ± 0.01	3.84 ± 0.11	0.92 ± 0.02	0.00 ± 0.03	0.00 ± 0.02	0.00 ± 0.02	0.00 ± 0.05	0	0.00 ± 0.05	0.00 ± 0.02	0.00 ± 0.03	0.00 ± 0.01	0.00 ± 0.01	0.00 ± 0.02	0.00 ± 0.01	-0.25 ± 0.05	0.00± 0.01	-0.19 ± 0.03
			ZabXc		10_0_16_3	68	117	-43m	1.01 ± 0.01	3.85 ± 0.08	0.91 ± 0.02	0.00 ± 0.01	0.00 ± 0.03	0.00 ± 0.03	0	0.00 ± 0.02	0.00 ± 0.01	0.00 ± 0.05	0.00 ± 0.05	0.00 ± 0.01	0.00 ± 0.01	0	0.00 ± 0.02	0.00 ± 0.02	-0.32 ± 0.04	0.00 ± 0.01
			ZabcXa		10_0_17_	110	126	3m (m_|_y)	1.01 ± 0.01	3.84 ± 0.11	0.92 ± 0.02	0.00 ± 0.01	0.00 ± 0.03	-0.02 ± 0.03	0.00 ± 0.01	0.00 ± 0.01	0.00 ± 0.05	-0.03 ± 0.03	-0.03 ± 0.04	0.00 ± 0.01	0.00 ± 0.01	0.00 ± 0.01	-0.25 ± 0.04	0.01 ± 0.03	0.00 ± 0.01	0.21 ± 0.03
N	2p	18	ZaXb		9_0__	16	17	m (m_|_y)	0.99 ± 0.00	5.02 ± 0.04	10.4 ± 0.02	0	-0.08 ± 0.02	-0.12 ± 0.02	0	0	0.01 ± 0.06	0.08 ± 0.03	0.07 ± 0.04	0	0	0	0.08 ± 0.02	-0.01 ± 0.01	0.04 ± 0.02	0.03 ± 0.01
			XabYa		9_1__	18	18	mm2 (2||x)	0.99 ± 0.00	5.03 ± 0.05	1.04 ± 0.02	0.00 ± 0.02	0	-0.14 ± 0.02	0.00 ± 0.06	0	-0.07 ± 0.03	0	0.08 ± 0.02	0.00 ± 0.01	0	0.00 ± 0.02	0	-0.03 ± 0.00	0	-0.09 ± 0.02
			ZabYa		9_2__	18	18	mm2 (2||z)	0.99 ± 0.00	5.03 ± 0.05	1.04 ± 0.02	0.00 ± 0.02	-0.14 ± 0.02	0	0	0.00 ± 0.06	0.11 ± 0.02	0	-0.01 ± 0.02	0.00 ± 0.02	0	0.00 ± 0.01	-0.06 ± 0.01	0	-0.08 ± 0.01	0
	3n, 3p	20, 56	ZaXb		9_4__	16 (3n) + 38 (3p)	17 (3n) + 39 (3p)	mm2 (2||z)	1.00 ± 0.00	5.02 ± 0.05	1.02 ± 0.04	0.00 ± 0.02	0.00 ± 0.02	-0.01 ± 0.02	0.00 ± 0.02	0.00 ± 0.01	-0.04 ± 0.02	0.00 ± 0.02	-0.06 ± 0.03	0.00 ± 0.01	0.00 ± 0.01	0	0.11 ± 0.02	0.00 ± 0.03	-0.08 ± 0.03	0.01 ± 0.02
			XabYa		9_5__	16 (3n) + 39 (3p)	17 (3n) + 39 (3p)	-6m2 (m_|_y)	1.00 ± 0.00	5.02 ± 0.05	1.02 ± 0.04	0.00 ± 0.02	0.00 ± 0.02	-0.01 ± 0.02	0.00 ± 0.02	0	0.08 ± 0.03	0.00 ± 0.02	0.00 ± 0.02	0.00 ± 0.01	0	0.00 ± 0.01	0	-0.01 ± 0.02	0.00 ± 0.01	-0.13 ± 0.03
	3n	20	ZabXc		9_7__	12	14	m (m_|_y)	0.99 ± 0.00	5.04 ± 0.05	1.04 ± 0.02	0.00 ± 0.01	-0.03 ± 0.03	-0.06 ± 0.03	0.00 ± 0.01	0.00 ± 0.01	-0.02 ± 0.02	0.07 ± 0.03	0.08 ± 0.02	0.00 ± 0.001	0.0 ± 0.01	0	-0.06 ± 0.02	0.03 ± 0.01	-0.10 ± 0.02	0.01 ± 0.01
			ZabcXa		9_8__	13 (only one rotation)		3m (3||z, m_|_y)	0.99 ± 0.00	5.04 ± 0.06	1.03 ± 0.02	0	-0.07 ± 0.03	0.00 ± 0.01	0	0.00 ± 0.01	0.12 ± 0.02	-0.01 ± 0.01	-0.01 ± 0.01	0	0.00 ± 0.01	0	-0.04 ± 0.01	0.00 ± 0.01	0	0.11 ± 0.01
	4n	9	ZaXb		24___	9	9	3m (3||z, m_|_y)	0.98 ± 0.01	5.20 ± 0.07	0.80 ± 0.02	0	0.00 ± 0.02	0.00 ± 0.02	0	0	0.00 ± 0.01	0	0.00 ± 0.01	0.00 ± 0.01	0	0	0.27 ± 0.01	0.00 ± 0.01	0.00 ± 0.01	0.22 ± 0.01
			XabYa		25___	9	9	mm2 (m||x)	0.98 ± 0.01	5.20 ± 0.07	0.80 ± 0.02	0.00 ± 0.02	0	0.00 ± 0.02	0.00 ± 0.01	0	0.00 ± 0.01	0	0	0	0	0	0.00 ± 0.01	0.27 ± 0.01	0	-0.20 ± 0.01
			ZabXc		26___	5	9	-43m	0.98 ± 0.01	5.20 ± 0.07	0.80 ± 0.02	0	0.00 ± 0.02	0.01 ± 0.01	0	0	0	0.00 ± 0.01	0.00 ± 0.01	0	0	0	0	0.00 ± 0.02	-0.35 ± 0.02	0.00 ± 0.01
			ZabcXa		27___	9	9	3m (3||z, m_|_y)	0.98 ± 0.01	5.20 ± 0.07	0.80 ± 0.02	0	0.00 ± 0.02	0.01 ± 0.02	0	0	0.00 ± 0.01	0	0.00 ± 0.01	0	0	0	-0.27 ± 0.01	-0.01 ± 0.01	0	0.22 ± 0.01
P	4n	11	ZabXc		36___	0	6	m (m_|_y)	0.94 ± 0.01	5.28 ± 0.06	1.05 ± 0.02	0	0.03 ± 0.05	-0.12 ± 0.03	0	0	0.03 ± 0.02	0.17 ± 0.03	-0.08 ± 0.06	0	0	0	-0.05 ± 0.03	-0.11 ± 0.03	-0.61 ± 0.04	0
			ZabXc		58___	0	4	m (m_|_y)	0.95 ± 0.00	5.31 ± 0.09	1.03 ± 0.01	0	-0.14 ± 0.03	-0.15 ± 0.03	0	0	0.04 ± 0.01	0.21 ± 0.01	0.12 ± 0.02	0	0	0	0.03 ± 0.01	-0.18 ± 0.02	-0.70 ± 0.01	0.00 ± 0.01
			ZaXb		64___	0	1	m (m_|_y)	0.95 ± 0.00	5.27 ± 0.00	1.04 ± 0.00	0	-0.04 ± 0.00	-0.04 ± 0.00	0	0	0	0.15 ± 0.00	0	0	0	0	0.44 ± 0.00	-0.10 ± 0.00	0.12 ± 0.00	0.39 ± 0.00
O	1p, 2p	44, 36	ZaXb (ZaXx), ZabYa		2_0_1_0	26 (1p) + 29 (2p)	26 (1p) + 29 (2p)	mm2 (m||z)	0.99 ± 0.00	6.16 ± 0.05	1.18 ± 0.03	0.00 ± 0.01	-0.09 ± 0.01	0	0	0.00 ± 0.02	0.00 ± 0.03	0	0.09 ± 0.03	0	0	0	-0.01 ± 0.02	0	-0.03 ± 0.01	0
	2p	36	ZaXb		2_0_3_1	14	17	m (m_|_y)	0.99 ± 0.00	6.18 ± 0.02	1.20 ± 0.02	0	-0.05 ± 0.01	-0.07 ± 0.01	0	0	-0.06 ± 0.01	0.06 ± 0.00	-0.04 ± 0.01	0	0	0	0.04 ± 0.00	0.00 ± 0.01	-0.02 ± 0.00	0.01 ± 0.00
	2p, 3n	36 , 2	XabYa	color only for 3n	2_3_0_	29 (2p) + 1 (3n)	29 (2p) + 1 (3n)	mm2 (2||x)	0.99 ± 0.00	6.18 ± 0.04	1.18 ± 0.03	0.00 ± 0.01	0	-0.09 ± 0.02	0.00 ± 0.02	0	0.08 ± 0.03	0	0.06 ± 0.01	0	0	0.00 ± 0.01	0	-0.02 ± 0.03	0	-0.04 ± 0.01
	3n	2	ZaXb		2_0_3_0	1	1	3m	0.99 ± 0.00	6.23 ± 0.00	1.20 ± 0.00	0.00	-0.03 ± 0.00	-0.04 ± 0.00	0.00	0.00	-0.03 ± 0.00	0.02 ± 0.00	-0.03 ± 0.00	0.00	0.00	0.00	0.05 ± 0.00	0.00	-0.01 ± 0.00	0.03 ± 0.00
			ZabXc		2_10__	1	1	m (m_|_y)	0.99 ± 0.00	6.23 ± 0.00	1.20 ± 0.00	0.00	-0.05 ± 0.00	-0.06 ± 0.00	0.00	0.00	0.00	0.07 ± 0.00	0.05 ± 0.00	0.00	0.00	0.00	-0.01 ± 0.00	0.00	-0.06 ± 0.00	0.01 ± 0.00
S	1p	6	ZaXb (ZaXx)		4___	6	6	mm2 (2||z)	0.98 ± 0.00	6.39 ± 0.03	1.00 ± 0.01	0	-0.05 ± 0.00	0	0	0	-0.13 ± 0.02	0.00 ± 0.01	0.08 ± 0.02	0	0	0	0.14 ± 0.00	0	-0.03 ± 0.01	0
	2p	14	ZaXb		2_4__	12	13	m (m_|_y)	0.98 ± 0.00	6.31 ± 0.05	1.0 ± 0.03	0	-0.03 ± 0.00	-0.04 ± 0.00	0	0	-0.07 ± 0.02	0.05 ± 0.01	-0.10 ± 0.03	0	0	0	0.20 ± 0.03	-0.09 ± 0.02	-0.05 ± 0.02	0.11 ± 0.01
			XabYa		2_5__	13	13	mm2 (2||x)	0.98 ± 0.00	6.30 ± 0.06	1.06 ± 0.03	0	0	-0.05 ± 0.00	0.00 ± 0.01	0	0.12 ± 0.03	0	0.05 ± 0.01	0.00 ± 0.02	0	0.00 ± 0.01	0	-0.12 ± 0.01	0	-0.21 ± 0.01
			ZabYa		2_6__	12	12	mm2 (2||z)	0.98 ± 0.00	6.31 ± 0.04	1.06 ± 0.03	0	-0.05 ± 0.00	0	0	0.00 ± 0.01	0.02 ± 0.01	0	0.13 ± 0.03	0.00 ± 0.01	0	0.00 ± 0.01	-0.11 ± 0.01	0	-0.23 ± 0.01	0
	3n	4	ZaXb		12_0__	3	3	m (m_|_y)	0.98 ± 0.00	6.16 ± 0.06	1.05 ± 0.01	0	-0.03 ± 0.01	-0.04 ± 0.01	0	0	-0.02 ± 0.02	0.03 ± 0.01	-0.02 ± 0.03	0	0	0	0.33 ± 0.03	-0.05 ± 0.02	-0.01 ± 0.06	0.24 ± 0.04
			XabYa		13_0__	3	3	mm2 (2||x)	0.98 ± 0.00	6.16 ± 0.06	1.05 ± 0.01	0.00 ± 0.01	0	-0.05 ± 0.01	0.00 ± 0.02	0	0.03 ± 0.03	0	0.04 ± 0.01	0.00 ± 0.01	0	0	0.00 ± 0.05	-0.29 ± 0.04	0	-0.27 ± 0.03
			ZabXc		14_0__	1	0 (rotation 45 is always an outlier in cluster 14_1__)	m (m_|_y)	0.98 ± 0.00	6.25 ± 0.00	1.06 ± 0.00	0	-0.04 ± 0.00	-0.04 ± 0.00	0	0	0.02 ± 0.00	0.07 ± 0.00	0.02 ± 0.00	0	0	0	-0.05 ± 0.00	-0.04 ± 0.00	-0.38 ± 0.00	0.09 ± 0.00
			ZabcXa		15___	3 (one rotation)		m (m_|_y)	0.98 ± 0.00	6.16 ± 0.06	1.05 ± 0.01	0	0.07 ± 0.01	-0.01 ± 0.01	0	0	0.09 ± 0.01	0.04 ± 0.01	0.02 ± 0.01	0	0	0	-0.28 ± 0.02	-0.09 ± 0.04	0.01 ± 0.02	0.31 ± 0.02
	4n	11	ZaXb		80_1__	0	9	m (m_|_y)	0.96 ± 0.00	5.95 ± 0.04	1.04 ± 0.01	0	0	0.01 ± 0.01	0	0	0.06 ± 0.02	0.03 ± 0.01	-0.02 ± 0.03	0	0	0	0.49 ± 0.01	-0.04 ± 0.02	0.03 ± 0.04	0.34 ± 0.01
			XabYa		81_0__	0	4	mm2 (2||x)	0.97 ± 0.00	5.98 ± 0.02	1.04 ± 0.01	0	0	-0.01 ± 0.00	0	0	-0.06 ± 0.01	0	-0.05 ± 0.01	0	0	0	0	-0.42 ± 0.01	0	-0.41 ± 0.01
			ZabXc		82_1__	0	9	m (m_|_y)	0.96 ± 0.00	5.95 ± 0.04	1.04 ± 0.01	0	0.00 ± 0.01	0.00 ± 0.01	0	0	0.01 ± 0.01	-0.07 ± 0.02	-0.02 ± 0.03	0	0	0.00 ± 0.01	-0.04 ± 0.02	0.13 ± 0.02	-0.57 ± 0.01	-0.01 ± 0.01
			ZabcXa		83_0__	0	5	m (m_|_y)	0.97 ± 0.00	5.97 ± 0.02	1.04 ± 0.01	0	0.00 ± 0.01	0	0	0	-0.06 ± 0.03	0.01 ± 0.01	0.03 ± 0.01	0	0	0	-0.41 ± 0.01	-0.08 ± 0.01	0	0.42 ± 0.01
F	1p	3	ZaXb		3___	3	3	cyl	0.99 ± 0.00	7.19 ± 0.02	1.24 ± 0.01	0	-0.06 ± 0.01	0	0	0	-0.10 ± 0.02	0	0.00 ± 0.01	0	0	0	0.01 ± 0.01	0	0	0
Cl, Br	1p	7	ZaXb (ZaXx)		5___	7	7	cyl	0.99 ± 0.00	7.23 ± 0.05	0.96 ± 0.03	0	-0.05 ± 0.01	0	0	0	-0.20 ± 0.01	0	0.01 ± 0.01	0	0	0	0.08 ± 0.02	0	0	0
unique atom types																										

Figure S12. The largest clusters in each group and local coordinate systems with corresponding cluster labels, mean values of multipoles and proposed symmetry.

Table S1. Exact definition of atom type C313b from the MATTS data bank.

```
ENTRY
 ID
  C313b
 COMMENT
  in UBDB2018: C313 splitted KJ
 NOI
  87
 ATOM DESCRIPTORS
#  central atom
  C1    CONNECTED_TO  C2,C3,C4        PLANARITY +  PLANAR_RING_WITH_PLANAR_ATOMS -       IN_3_MEMBER_RING - IN_4_MEMBER_RING -
#  1-st neighbors
  C2    CONNECTED_TO  C1,X,X          PLANARITY +  PLANAR_RING_WITH_PLANAR_ATOMS *       IN_3_MEMBER_RING * IN_4_MEMBER_RING *
  C3    CONNECTED_TO  C1,X,X,X        PLANARITY -  PLANAR_RING_WITH_PLANAR_ATOMS -       IN_3_MEMBER_RING * IN_4_MEMBER_RING *
  C4    CONNECTED_TO  C1,X,X,X        PLANARITY -  PLANAR_RING_WITH_PLANAR_ATOMS -       IN_3_MEMBER_RING * IN_4_MEMBER_RING *
 LOCAL COORDINATE SYSTEM
  Z C2 X C3 R
 SYMMETRY
  mm2
 PARAMETER MODIFICATION DATE
  Fri Dec  3 10:28:39 2021
 MULTIPOLE MODEL PARAMETERS
  PVAL        4.13(10) KAPPA      0.9929(64) KPRIM       0.872(24)
  PLMS   1  0     0.082(24) PLMS   2  0     0.166(28) PLMS   2  2     0.123(26) PLMS   3  0     0.215(32)
  PLMS   3  2    -0.241(34) PLMS   4  4     0.030(10)
```

Table S2. Representation of zeroed P_{lm} values.

Symmetry	Coordinate axes	P_{1-1}	P_{10}	P_{11}	P_{2-2}	P_{2-1}	P_{20}	P_{21}	P_{22}	P_{3-3}	P_{3-2}	P_{3-1}	P_{30}	P_{31}	P_{32}	P_{33}
$\bar{4}3m$	$\bar{4} \parallel z$	0	0	0	0	0	0	0		0	0	0		0		0
$\bar{6}m2$	$\bar{6} \parallel z, m \perp y$	0	0	0	0	0		0	0	0	0	0	0	0	0	
$\bar{6}m2$	$\bar{6} \parallel z, m \perp x$	0	0	0	0	0		0	0		0	0	0	0	0	0
$cyl*$	$\infty \parallel z$	0		0	0	0		0	0	0	0	0		0	0	0
$3m$	$3 \parallel z, m \perp y$	0		0	0	0		0	0	0	0	0		0	0	
$3m$	$3 \parallel z, m \perp x$	0		0	0	0		0	0		0	0		0	0	0
$mm2$	$2 \parallel z$	0		0	0	0		0		0	0	0		0		0
$mm2$	$2 \parallel y$		0	0	0	0		0			0		0	0	0	0
$mm2$	$2 \parallel x$	0	0		0	0		0		0	0	0	0		0	
m	$m \perp z$		0			0		0			0		0		0	
m	$m \perp y$	0			0	0				0	0	0				
m	$m \perp x$			0	0			0			0			0		0
no	any															

cyl * - cylindrical symmetry, i.e. where only one axis of rotation $\infty \parallel z$ is present (∞ is an axis with any possibility of fold)

no = no symmetry, e.g. point group 1

Table S3. Statistical analysis of the topology clustering results.

Cluster size	No. of atom types	No. of clusters	% of all clusters	***Central atom***	No. of atom types	No. of clusters	***No. of first neighbors***	No. of atom types	No. of clusters
1	305	305	71.26	***C***	156	156	***2***	4	4
							3	87	87
							4	65	65
				N	49	49	***1***	1	1
							2	9	9
							3	30	30
							4	9	9
				O	46	46	***1***	18	18
							2	26	26
							3	2	2
				S	26	26	***1***	6	6
							2	7	7
							3	4	4
							4	9	9
				P	12	12	***4***	11	11
							6	1	1
				H	9	9	***1***	8	8
							2	1	1
				F	3	3	***1***	3	3
				Cl	2	2	***1***	1	1
							4	1	1
				Br	2	2	***1***	2	2
2	134	67	15.65	***C***	84	42	***3***	54	27
							4	30	15
				N	30	15	***2***	6	3
							3	24	12
				O	6	3	***2***	6	3
				S	6	3	***2***	4	2
							4	2	1
				H	4	2	***1***	4	2
				Cl	4	2	***1***	4	2
3	87	29	6.78	***C***	54	18	***3***	36	12
							4	18	6

				N	15	5	***2***	3	1
							3	12	4
				O	12	4	***1***	12	4
				S	3	1	***2***	3	1
				H	3	1	***1***	3	1
4	64	16	3.74	***C***	44	11	***3***	28	7
							4	16	4
				O	8	2	***1***	4	1
							2	4	1
				H	8	2	***1***	8	2
				N	4	1	***3***	4	1
5	30	6	1.40	***C***	15	3	***3***	15	3
				O	10	2	***1***	10	2
				H	5	1	***1***	5	1
6	24	4	0.93	***C***	18	3	***3***	12	2
							4	6	1
				N	6	1	***3***	6	1
7	7	1	0.23	***C***	7	1	***3***	7	1
Total	***651***	***428***	***100***		***651***	***428***		***651***	***428***

Appendix 1. The computational details of rotation of local coordinate system

The procedure of rotating local coordinate systems contained several steps. Firstly, the MATTS2021 data bank entries were divided into individual files and then, their copies with several options for the local coordinate system choice were generated using local scripts. The symmetry filter was set to "no", thus no filtering by symmetry was applied to P_{lm} parameters. Lastly, the program *bankMaker* v0.016 from the *DiSCaMB* library was run with files containing modified entries from the MATTS2021 data bank, and files containing coordinates and multipole model parameters for 2516 model molecules from the MATTS2021 collection as inputs[1]. The *bankMaker* is a utility program within the newest version of *DiSCaMB* library. The program generated new P_{lm} values as an output. Atom types for which at least one P_{lm} parameters appeared to have sample standard deviation larger than 0.05 e were assigned a flag "inconsistent".

Appendix 2. The computational details of topology clustering

To obtain an extended tree from topology clustering, a few steps were necessary, all of them required local scripts with the use of graph description language DOT, as well as bash and Python programming languages. Firstly, the MATTS2021 data bank entries were divided into individual files using bash language. From each entry (one atom type) several pieces of information were automatically extracted and added to a new dataset: ID of an atom type, chemical element of the central atom, number and types of first neighbors, planarity properties (planarity, planar rings with planar atom, size of rings), symmetry from the data bank, sum of second neighbors and their types, and final label created for topology clustering purposes. Then, using bash language again, a loop through all the dataset entries generated a .py script with parent-child relations that are a basis of a tree, written in a format suitable to use anytree 2.8.0 Python package[2]. This script was then

executed using Spyder 4.1.4[3] and a .dot file (format of the graph description language DOT) was created. This file could already be used to generate an extended tree, but we edited it and included shorter labels for ”parents” and “children”, and added background color to show planarity. This was done automatically using local bash scripts. Then, from a final .dot file an extended tree was generated using Graphviz[4] (an open-source visualization software for Windows) and Windows command line. In the DOT language many output formats are available, we chose .png to generate an image.

Appendix 3. The computational details of electron density clustering

The process of electron density clustering was divided into two steps: data preprocessing and actual clustering. The MATTS2021 data bank entries were divided into individual files using bash language. Then, ID of an atom type and multipole model parameters (κ, P_{val}, κ' and P_{lm}) were automatically extracted from each entry and added to a new dataset in a .csv format. This dataset was imported into Python using pandas 1.0.5[5] library. Also, other standard data science libraries were used: NumPy v1.19.0[6] (for data manipulation), Matplotlib 3.2.2[6] (for plotting), and scikit-learn 0.23.1[7] (for DBSCAN algorithm). For finding Eps parameters, scikit-learn.neighbors.NearestNeighbors and KneeLocator[8] libraries for Python were used.

Appendix 4. Hydrogen atom types

Hydrogen atom types belong in the 1x group with the exception of H122 which is a middle H in the oxonium ion $H_2O_5^+$ and thus, belongs to the 2x group (see Figure 11). Atom types from the 1x group divide into two main clusters (0___ and 1___) in line with expectations from their chemical properties, the separating factor being the type of a first neighbor indicating whether said hydrogen

is polar or nonpolar. In those clusters, P_{10} and P_{20} multipoles have positive values, the rest are zero as expected (they were never refined in the model molecules). It is noteworthy that H120 (hydrogen in the hydronium ion H_3O^+) is excluded from the 1___ cluster despite having similar P_{10} and P_{20} values, presumably due to its significantly lower P_{val} and higher κ values. As expected, H122 creates a separate cluster (6___) with a positive P_{20}. All hydrogen type clusters exhibit cylindrical symmetry, as expected. For all hydrogen type clusters sample standard deviations of almost all MM parameters are not exceeding the desired values (0.1 for P_{val} and κ', 0.01 for κ, and 0.05 for P_{lm}). The only exception is κ for polar hydrogen type cluster (1___) having sample standard deviation twice larger than desired, indicating this cluster may need to be further divided before general hydrogen atom types are to be defined on the basis of this analysis. There are no atom types with inconsistent multipole parameters among hydrogen atom types.

Appendix 5. Carbon atom types

Considering that carbon atom types make up the majority of the MATTS data bank and multiplying each atom type by the number of all possible rotations, it is challenging to determine clear carbon type categories. However, some trends can be observed and graphical representation of clustering of carbon atom types is shown in Figures S5 and S6. In the 2x group there are four carbon atom types, all of them detached from other carbon type groups and divided into two categories already on the first level of the clustering. The first category has three elements: atom types C202, C203a and C203c with non-zero P_{30}, and P_{val} close to 4 e. Due to the flip of sign for the P_{10} multipole, two clusters are created and define the first category: one for rotations with positive P_{10} (cluster 7___), and one for rotations with negative P_{10} (cluster 8___). The second category in the carbon 2x group has only one element: atom type C201 with P_{30} equal 0 e and P_{val}

equal 4.474 e, which is labeled as outlier on the first level of clustering. Chemically, C202, C203a, and C203c are from unsaturated aliphatic hydrocarbons, whereas C201 is a carbon from the nitrile group. All the two carbon 2x clusters exhibit cylindrical symmetry, as expected. For all the two clusters, sample standard deviations of almost all MM parameters are acceptable, only for P_{10} it is slightly too large (ssd = 0.06 e). Two atom types from the 2x group received “inconsistent” label for at least one of rotation: C202 and C203c.

The 3n, 3p, and 4n groups of carbon atom types stay together until the third level of clustering, where the main separation by groups, corresponding to the local coordinate systems available for each group (ZaXb and XabYa for 3p, ZaXb, XabYa, ZabXc, ZabcXa for 3n and 4n), occurs. The 3n group contains only one atom type - C3a2, which is almost planar and seems similar to types from the 3p group in all local coordinate systems common for these two groups. With the remaining two other types of local coordinate system associated with the 3n group, the C3a2 type creates its own unique cluster (11___ for ZabXc) or becomes an outlier (ZabcXa). In groups 3p and 4n, large clusters for each coordinate system are created and they contain the majority of carbon atom types that for given conditions of the analysis (Eps, levels of clustering, sample standard deviation etc.) are similar. Inconsistencies for some of the rotations are present for 64 from 241 atom types (26.8 %) in the 3p group, and 111 from 132 atom types (84.1 %) in the 4n group, suggesting that many of the rotations may not be optimal for MM parameters averaging.

At the fourth level of clustering for the carbon 3p group, the majority of atom types cluster with each other in the XabYa system forming cluster labeled 10_1_0_0. The cluster contains 236 atom types out of 241 present in the carbon 3p group, and the one type from the carbon 3n group. For the ZaXb system, a slightly smaller cluster is generated, labeled 10_0_0_0 and contains 228 atom types. However only 191 of the types have all their ZaXb type of rotations in the cluster. Among

atom types which appear in the 10_1_0_0 cluster but not in the 10_0_0_0 cluster there are a few distinct atom types, e.g. C379. Topologically, C379 is the only atom type in the MATTS data bank that simultaneously belongs to 5-membered ring and has two nitrogen and one oxygen as the first neighbors. All rotations of C379 in the XabYa system have similar parameters and belong to the large cluster with label 10_1_0_0, but in the ZaXb they differ – those rotations where the Z axis is defined in the direction of an oxygen atom belong to the large cluster with label 10_0_0_0, and those with the Z axis defined in the direction of a nitrogen atom, separate themselves from the main cluster due to the change in values and signs of P_{31} and P_{33} multipoles. A similar situation occurs in pairs of atom types that detach from the main 10_0_0_0 cluster, for example atom types C555b and C786a. Topologically those atom types differ slightly, as they both belong to 5-membered rings but C555b has N, N, C, and C786a has S, N, C as first neighbors. Another pair of atom types C008 and C521b in the XabYa system belongs to the large cluster 10_1_0_0, however in the ZaXb system creates their own clusters. The explanation may arise from topological properties – both C008 and C521b are carbons covalently bonded with two carbon and one sulfur atoms, and have 6 second neighbors.

For both clusters, 10_0_0_0 and 10_1_0_0, the mean values of P_{lm} suggest that atom types belonging to them have the highest possible local symmetry observed in a particular, local coordinate system, $\bar{6}$m2 (m⊥y) for the XabYa and mm2 (2‖z) for the ZaXb system. The multipoles with the highest absolute values (negative P_{33} for XabYa, positive P_{30} and negative P_{32} for ZaXb) confirm that the shape of electron density for that clusters follows the sp2 hybridization. However, several P_{lm} which should vanish within the above symmetries averaged to zero values with large sample standard deviations (ssd = 0.07 e), also many symmetries allowed P_{lm} to have large sample standard deviations. All this clearly shows that these two clusters should be further divided.

Additionally, at each level of clustering starting from the second one, unique carbon atom types are observed. Five unique atom types, C309a, C382c, C569T, C783, and C797, from the carbon 3p group remain outside the main 10_1_0_0 cluster. The most noticeable one is C783 which has all its ZaXb rotations in a separate 10_14__ cluster, and all XabYa rotations in the separate 10_15__ cluster. It is reasonable as C783 describes the central carbon in the SO_2-C(C)-SO_2 chemical group and is unlike any other atom type described in the MATTS2021 data bank. Other unique atom types are: C309a, C569T, C797, C382c. The C309a atom type has the lowest P_{val} comparing to all atom types from the 3p group. The C569T atom type is connected with 1 carbon and 2 sulfur atoms and belongs to a 5-membered ring. The C797 atom type is connected to 3 other carbons and belongs to both 4-membered and 6-membered (benzene) rings.

For the carbon 4n group, containing 132 atom types, the situation is similar to the carbon 3p group. In each of the four local coordinate system types possible, a large cluster with majority of the same atom types is present. In the ZaXb system the 10_0_16_2 cluster has 117 atom types with at least one rotation, or 84 atom types with all their rotations in it, in the XabYa system – 131 atom types with at least one rotation or 119 atom types with all their rotations in cluster 10_1_0_1, in the ZabXc system – 117 atom types with at least one rotation or 68 atom types with all their rotations in cluster 10_0_16_3, and in the ZabcXa system – 126 atom types with at least one rotation, or 110 atom types with all their rotations in it (cluster 10_0_18_). Distinct atom types present in the largest cluster with at least one rotation in the XabYa system, but absent in other systems (specified in the brackets) are: C432b, C882 (ZaXb); C401, C410, C433, C756 (ZabXc); C408, C417a, C435, C463, C472, C798, C799b (ZaXb, ZabXc); C4111, C7902, C953b (ZaXb, ZabXc, ZabcXa); C953c (ZaXb, ZabcXa); C799 (ZabcXa). Those distinct atom types stand out in some way e.g., C799b, C953a, and C953c belong to a 3-membered ring, C791, C793, C798, and

C7902 belong to 4-membered rings. Apart from the ring membership, in some types rare and specific combinations of first neighbors occur: C4691 is connected to Br and three C atoms, C882 to C and three F atoms, C452 to S, N, and two H atoms, C442 to S, N, and two C atoms, C432b to P, C, and two H atoms, C4111 to N, and three C atoms, and finally C782 is a carbon atom from SO_2-CH(C)-SO_2 chain. There are also groups such as: C756 and C889 (both connected to three F and one C atoms), C455 and C456 (both connected to two F and two C atoms, C7911 and C7931 (both in 4-membered rings, connected to two C and one H atoms, as a fourth neighbor C7911 has a N atom, and C7931 has an O atom), C435 and C953b (carbons from an epoxide 3-membered ring), C792, C7921 and C794 (4-membered rings, connected to H, two C, and either N or O atoms), C417a, C463 and C472 (connected to two or three C, and one or two N atoms), C4041, C404D, C799 and C998 (all are in 3-membered rings, at least two C neighbors, the remaining two are C or H atoms).

The sample standard deviations of MM parameters of the largest clusters of carbon 4n group usually do not exceed the desired values or exceed them only slightly. However, the 4n carbon group is the one which has also large percentage of atom types labeled with the inconsistence flag.

In all the clusters, P_{lm} obey the maximum symmetry possible for a particular coordinate system and agree with the sp3 shape of electron density (positive P_{30} and P_{33} for ZaXb, negative P_{31} and P_{33} for XabYa, negative P_{32} for ZabXc, and negative P_{30} and positive P_{33} for ZabcXa). Similarly like with the 3p group, the XabYa coordinate system allows the largest cluster of similar atom types to form. The most restrictive is the ZabXc system, suggesting that only 68 atom types for which all rotations belongs to the 10_0_16_3 cluster truly have $\bar{4}3m$ symmetry. There is only one unique atom type which does not appear in any of the above large clusters, it is C840.

Appendix 6. Nitrogen atom types

Nitrogen atom types may belong to 2p, 3n, 3p, or 4n groups, there is also one atom type in the 1x group: N101. The 2p group, containing 18 atom types, is fairly unfluctuating with atom types divided by local coordinate systems: ZaXb (two possible rotations) in cluster 9_0__, XabYa (two possible rotations) in cluster 9_1__ and ZabYa (two possible rotations) in cluster 9_2__. In the ZaXb system almost all N atom types are clustered together, except N207a and N207b (nitrogen atom types with two nitrogen atoms as the first neighbors, the only 2p nitrogen type with two neighbors other than carbon or nitrogen). They detach themselves from other 2p N atom types in the ZaXb system but do not cluster with each other, and according to definitions introduced in paragraph 3.3.2, are named 'distinct' and marked in Figure S7 by lighter background. All the clusters show the highest possible symmetry and sample standard deviations of their parameters do not exceed desired values. It seems it would be possible to define a general atom type for the nitrogen 2p group. There is only one atom type with inconsistent parameters: N203a.

Ca. 70 % of 3p nitrogen atom types cluster together with each other in clusters 9_4__ (ZaXb system) and 9_5__ (XabYa system). Each of the clusters also contain the majority of 3n nitrogen atom types. Both clusters contain the same atom types, they only differ because of different rotation type. Coexistence of atom types from two groups, 3n and 3p, in one cluster shows the significance of using 'purple' local coordinate systems (shown previously in Figure 2) designed to allow such situation. Those clusters were not further divided as the standard deviation for each P_{lm} was smaller than the accepted limit of 0.05 e. Only 4 of 20 atom types from the 3p group have inconsistent parameters at some rotations: N327, N335b, N3592, N729. There is no inconsistency among the 3n group.

Many nitrogen atom types from the 3n group also form large clusters in the remaining two coordinate systems, cluster 9_7__ in ZabXc and cluster 9_8__ in ZabcXa. Almost all nitrogen atom types in cluster 9_7__ appears also in previously mentioned cluster 9_5__. The P_{lm} values of clusters 9_4__ and 9_5__ indicate the clusters contain atom types having sp2 hybridization (negative P_{33} and vanishing P_{31} for XabYa, positive P_{30}, negative P_{32} and vanishing P_{33} for ZaXb). Though the absolute values of P_{lm} are twice or more smaller than for sp2 carbon atom types in clusters 10_0_0_0 and 10_1_0_0.

Sp2 hybridization for nitrogen types from the 3p group follows chemical intuition, however this is somehow unexpected to see that majority of nitrogen atom types from the 3n group also seem to have electron density resembling more sp2 than sp3 hybridization. Deeper investigations are required to better understand possible causes of this observation (specific geometry, not unique enough definition of coordinate system, etc.) Clusters 9_7__ and 9_8__ containing only 3n nitrogen types have P_{lm} values which do not fully follow sp3 hybridization. In the ZabXc system the P_{32} has the largest absolute value but the P_{30} has not disappeared, whereas in the ZabcXa system, the P_{33} is not balanced well by the P_{30}, the latter being ca. twice smaller. This is most probably because electron density of lone electron pair at nitrogen atoms has to be solely described by that nitrogen atom multipole functions, whereas electron density of covalent bonds is usually described by multipolar functions of both, central nitrogen atom and its covalent neighbors. Thus multipolar functions contributing to lone electron pair descriptions have to have higher populations.

As usual, there are some atom types that never appear in the largest clusters of the nitrogen 3p/3n group. For example, in cases of some rotations, N308 and N449 atom types from the 3n group seem to be similar to each other than to the others and differentiate themselves from other 3n types

(see i.e. clusters from 9_10__ to 9_19__). In the 3p group, this situation happens for atom types N318, N335a, N452, and N453 as well as with a group made from N322a, N323a, and N325a atom types (cluster 9_57__) or smaller one with N3592 and N459 types (cluster 9_29__). Another unique group shows the importance of topology properties as it contains all 3p atom types with N and O as first neighbors - N998 and N998a (clusters from 9_43__ to 9_48__). There are also unique atom types such as N334, N339, N312, N332, or N3591.

The nitrogen 4n group creates only 5 clusters, following five possible types of coordinate systems and each cluster contains all atom types form this group with one exception as outlined below: 24___ (ZaXb system), 25___ (XabYa system), 26___ (majority of rotations from the ZabXc system), 27___ (ZabcXa system) and 28___ (some of rotations from the ZabXc system). The presence of two clusters for the ZabXc system is due to the incorrect flip of the sign for P_{32} multipole in some rotations for atom types: N401a, N401b, N402, N403. Within each cluster, MM parameters have their sample standard deviations smaller than the desired threshold, whereas their mean values follow clearly the electron density shape of the sp3 hybridized atoms and fulfill requirements for the highest possible symmetries to be seen in particular coordinate system. Only one atom type from the 4n group has inconsistent parameters for some of the rotations: N404.

Appendix 7. Oxygen atom types

Oxygen atom types belong either in the 1p, 2p, or 3n group and cluster in a complicated way, which can be seen in Figure S8. None of the oxygen atom types received label of being inconsistent. 44 atom types from the 1p group are divided into two main clusters that differ from each other in terms of the P_{22} and P_{val} parameters. The first cluster, labeled 2_0_1_0, includes 26 atom types from the 1p group in ZaXb system for which P_{10} has a negative and P_{22} has a mostly

positive value, but also includes atom types from the 2p group in the ZabYa system. Mean value of P_{val} equals 6.163 e, or 6.126 e when excluding the 2p group. The second cluster of oxygen 1p types (7 atom types, cluster 2_0_2_0) includes atom types for which still P_{10} has a negative value but P_{22} is zero and P_{val} is much larger, with mean value 6.273 e. The rest of the atom types from the 1p group make either distinct clusters (O101, O189) or occur in pairs (O121 and O1999, O370 and O122f, O113 and O998, O371 and O372).

26 out of 36 oxygen atom types from the 2p group cluster together in the ZabYa system (cluster 2_0_1_0, all rotations of the ZabYa system), except for O001, O210, O211b, O257, O258, O272, and O793 atom types. As mentioned above, this is the same cluster in which the majority of oxygen 1p types are. Co-clustering of 1p and 2p oxygen types from two different coordinate systems can be understood while looking at pictures visualizing local coordinate systems (see Figure 3 or Figure S1), remembering that 2p is derived from 4n with lone electron pairs in place of atoms c and d, and 1p is derived from 3p with lone electron pairs in place of atoms b and c. Such a combination of number of first neighbors and local coordinate system orientation allows to orient the system the same way with respect to lone electron pairs. Existence of that mixed clusters suggest that these oxygen types are not contributing any electron density to bonding with its covalent neighbors, but they do contribute to lone electron pair density. Nevertheless, departure of oxygen types from asphericity is relatively small, P_{lm} values are much smaller than observed for carbon types.

Same set of oxygen 2p atom types but with XabYa rotation create alternative cluster, 2_3_0_, but this time no oxygen types from 1p group are included in that cluster due to not having such rotations. Instead, oxygen type from the 3n group joined the cluster. For the ZaXb system, however, the 2p group of oxygen types divide into smaller clusters, the largest one, 2_0_3_0

contains only ca. 40% of atom types. Sample standard deviations for all MM parameters for all large clusters of oxygen types are below the threshold values. The mean values of the P_{lm} parameters in the case of cluster 2_0_3_1 and 2_3_0_ points towards sp3 hybridization of 2p and 3n oxygen types belonging to them, however here dipole and quadrupole functions have larger populations than octupoles, opposite to what was observed for carbon clusters. Again, departure from ideal set of populations typical for the sp3 hybridization results from the need to describe the lone electron pairs.

The unique atom types from the 2p group are O001 (an oxygen atom from an H2O molecule), O210, and O272, the latter being only one oxygen atom type that belongs to a 3-membered ring. Lastly, there are only two atom types that belong to the 3n group: O324 ($H_5O_2^+$ in oxonium) that is unique, and O323 (H_3O^+) that in the XabYa system shows similarity to the 2p group being in the 2_3_0_ cluster.

Appendix 8. Phosphorus atom types

Phosphorus atom types belong either in the 4n or 6n group, the latter including only one atom type: P601, which naturally is unique. The remaining eleven types can be divided into two groups that are heavily differentiated by the sign and values of the multipoles already on the first level of clustering (see clusters from 29___ to 79___). One group includes atom types P401, P409a, P409c, and P410. The second group includes atom types: P402, P403, P404a, P405, P407, and P408. Both of them are shown in Figure S9. Interestingly, P404b is an undecided atom type that can be similar to either of the groups with the change of coordinate systems. The fact that even at first level of clustering phosphorous types are highly divided (do not form any large cluster) might be because in general P_{lm} values of phosphorous types are among the highest observed for all atom types, and the *Eps* value optimal for entire dataset is already too small for phosphorus types to keep them

together. Also, each 4n phosphorus atom type has inconsistencies among multipole parameters for many rotations.

Appendix 9. Sulfur atom types

Visualization of density clustering results on a general tree for sulfur atom types is shown in Figure S10. All six sulfur atom types from the 1p group create one cluster (4___). Sample standard deviations of MM parameters for the cluster are small and acceptable. From the mean P_{lm} values it is clear that the types do not have cylindrical symmetry, the P_{22} has not averaged to zero, though it has smaller absolute value than P_{20} and P_{30}.

The situation is more complicated for the remaining groups of the sulfur atom types. In the 2p group most atoms are together in clusters 2_4__ (ZaXb system), 2_5__ (XabYa system) and 2_6__ (ZabYa system) with the exception of S213 and S220, both having C and N as first neighbors. S213 is similar to the main group in ZaXb and XabYa systems but in the ZabYa system, it becomes more like S220. Again, sample standard deviations of parameters are acceptable. Symmetries resulting from the P_{lm} values are the highest possible to be easily spotted within each of the coordinate system. One can conclude that sulfur atom types belonging to the above cluster have electron densities fulfilling the sp3 hybridization, though electron density lobes originating from sulfur atom and directed to the covalent partner are different from those directed to the positions of the two lone electron pairs. For sure, sulfur types are by far more aspherical than oxygen types, as analogous multipoles of sulfur types have much higher populations. In the 2p group, there is only one atom type with inconsistent multipole populations: S220.

Next, there is the 3n group that contains only four sulfur atom types and three of them (S320, S399, S001) cluster together in three of four possible local coordinate systems: ZaXb, XabYa,

ZabcXa (clusters 12_0__, 13_0__, and 15_0__). In the ZabXc system each of them creates separate clusters. Clusters 12_0__ and 13_0__ have acceptable values of sample standard deviations of their parameters, and as for sulfur 2p clusters, almost follow the requirement for the sp3 hybridization. Regarding symmetries resulting from the P_{lm} values, the situation is strange. For ZabcXa system, the symmetry is much lower than the maximal possible, m instead of 3m, though the multipoles violating 3m symmetry have smaller populations than the one fulfilling it. For XabYa system, the symmetry is too high. Apparently the system focuses too much on only two neighboring atoms, not describing properly contributions from the third neighbor and lone electron pair. There is no inconsistency observed among 3n sulfur atom types.

Lastly, in the 4n group (eleven atom types, clusters from 80_0__ onwards) there are two clear unique types, i.e. S411 and S442. At least one rotation of the remaining types appears in the largest cluster for each coordinate system, although all the rotations are never present in one cluster. The four largest clusters of sulfur 4n types have acceptable values of sample standard deviations of their parameters, and as for sulfur 2p and 3n clusters, almost follow the requirement for the sp3 hybridization. Here however no lone electron pairs are expected, all the four lobes are directed to chemical bonds. Apparently the bonds are not equivalent and the observed symmetry is mm2 (not $\bar{4}$3m nor 3m), though again the multipoles violating the symmetry have much smaller absolute populations with respect to those the most populated. In fact, for sulfur atom types multipoles dominant in each group have significantly larger absolute values than the rest of the atom types, with exception for phosphorous types which have the highest. 10 of 11 sulfur atom types from the 4n group have inconsistent multipole parameters for some of the rotations.

Appendix 10. Chlorine, bromine, and fluorine atom types

The least numerous atom types present in the MATTS2021 data bank are halogens: only three F, two Br, and six Cl. All of them belong to the 1p group, except for the Cl04 atom type that corresponds to the chlorine atom in the perchlorate ClO_4^- ion and thus belongs to the 4n group and differs from other chlorine atom types. All fluorine atom types cluster together (30___), as shown in Figure S11. Similarly, bromine and chlorine atom types cluster together, which suggests their chemical similarity (5___). All the halogen clusters have low sample standard deviations for density parameters, and all of them exhibit cylindrical symmetry. Also, there is no inconsistency in multipole parameters for any single atom type.

REFERENCES

(1) Chodkiewicz, M. L.; Migacz, S.; Rudnicki, W.; Makal, A.; Kalinowski, J. A.; Moriarty, N. W.; Grosse-Kunstleve, R. W.; Afonine, P. V.; Adams, P. D.; Dominiak, P. M. DiSCaMB : A Software Library for Aspherical Atom Model X-Ray Scattering Factor Calculations with CPUs and GPUs. *J. Appl. Crystallogr.* **2018**, *51* (1), 193–199. https://doi.org/10.1107/S1600576717015825.

(2) Any Python Tree Data. **2016**. GitHub repository. https://github.com/c0fec0de/anytree/blob/master/docs/index.rst (accessed January 20th, 2020)

(3) Raybaut, P. Spyder-documentation. *Available Online at: Pythonhosted. Org*. **2009.** (accessed April 30th, 2021)

(4) Ellson, J.; Gansner, E.; Koutsofios, L.; North, S.; Woodhull, G. Graphviz – Open Source Graph.Pdf. **2000**, 3–4. https://doi.org/10.1007/3-540-45848-4_57

(5) McKinney, W. Data structures for statistical computing in python. In *Proceedings of the 9th Python in Science Conference*. **2010**, *445*, 51–56. https://doi.org/10.25080/Majora-92bf1922-00a

(6) Harris, C. R.; Millman, K. J.; van der Walt, S. J.; Gommers, R.; Virtanen, P.; Cournapeau, D.; Oliphant, T. E. Array programming with NumPy. *Nature*, **2020**, *585*, 357–362. https://doi.org/10.1038/s41586-020-2649-2

(7) Hunter, J. D. (2007). Matplotlib: A 2D graphics environment. *Computing in Science & Engineering*. **2007**, *9*(3), 90–95. https://doi.org/10.1109/MCSE.2007.55

(8) Pedregosa, Fabian. Scikit-Learn: Machine Learning in Python. *J. Mach. Learn. Res.* **2011**, *12*, 2825–2830. https://arxiv.org/abs/1201.0490

(8) Arvai, K. Kneed. **2017.** GitHub repository. https://github.com/arvkevi/kneed (accessed March 15th, 2021)

Separate files:

'density-clustering.xlsx' – an Excel file with all the obtained results and the one used for DBSCAN.; **'topology-clustering-extended-tree.png'** – an image of an extended tree that shows topology data in the form of hierarchical structure. For coloring, see the legend in Figure S2.; a compressed folder with bash and Python scripts, including: **'rotate.sh'** – a bash script for rotation of the local coordinate system, '**create-dataset-for-topology-clustering.bash'** – a bash script for generating the dataset for the topology clustering, **'create-dataset-for-electron-density-clustering.bash'** – a bash script for generating the dataset for the electron density clustering, **'PART1_topology-clustering.bash'** and **'PART2_topology-clustering.bash'** – a two-part bash

script for the topology clustering, **‘electron-density-clustering.py’** – a Python script for the electron density clustering.
